# Identification of cis-regulatory modules in promoters of human genes exploiting mutual positioning of transcription factors

**DOI:** 10.1093/nar/gkt578

**Published:** 2013-08-02

**Authors:** Soumyadeep Nandi, Alexandre Blais, Ilya Ioshikhes

**Affiliations:** ^1^Ottawa Institute of Systems Biology, University of Ottawa, Ottawa, Ontario K1H 8M5, Canada and ^2^Department of Biochemistry, Microbiology and Immunology, University of Ottawa, Ottawa, Ontario K1H 8M5, Canada

## Abstract

In higher organisms, gene regulation is controlled by the interplay of non-random combinations of multiple transcription factors (TFs). Although numerous attempts have been made to identify these combinations, important details, such as mutual positioning of the factors that have an important role in the TF interplay, are still missing. The goal of the present work is *in silico* mapping of some of such associating factors based on their mutual positioning, using computational screening. We have selected the process of myogenesis as a study case, and we focused on TF combinations involving master myogenic TF Myogenic differentiation (MyoD) with other factors situated at specific distances from it. The results of our work show that some muscle-specific factors occur together with MyoD within the range of ±100 bp in a large number of promoters. We confirm co-occurrence of the MyoD with muscle-specific factors as described in earlier studies. However, we have also found novel relationships of MyoD with other factors not specific for muscle. Additionally, we have observed that MyoD tends to associate with different factors in proximal and distal promoter areas. The major outcome of our study is establishing the genome-wide connection between biological interactions of TFs and close co-occurrence of their binding sites.

## INTRODUCTION

Gene regulation in higher organisms is affected by multiple specific proteins called transcription factors (TFs). The human genome exhibits a spectacular example of sophisticated transcriptional regulation. TFs bind specifically to short DNA sequence motifs [TF binding sites or (TFBSs)] often clustered together.

The spatial combination of multiple such binding sites or elements is non-random in nature and forms Cis-regulatory modules (CRMs) (1–3). The interplay between the TFs that compose the CRMs plays an important role in gene regulation in eukaryotes (4). This is underscored by the fact that ∼25 000 human genes are controlled by <2000 sequence specific DNA-binding TFs (5,6). Eukaryotic gene expression is controlled by a number of different TFs bound to DNA as CRM combinations. The study by (7) shows that regulatory regions contain multiple functional binding sites. The CRMs retain their ability to regulate genes *in vitro* and lose the ability if the binding is disrupted by either eliminating a certain TF or its binding site (7). Similarly (8–10) showed that the association between TFs is a key to generating muscle-specific expression.

For computational analyses, TFBSs are often represented by position weight matrices (PWM) also known as position-specific scoring matrix, which can be used to detect TFBSs in genomic sequences (11–19). There exist some frequently used databases of TFs and their binding motifs, e.g. Jaspar and TRANSFAC.

The binding sites (or motifs) for particular TF are the building blocks/components of the CRM. The binding sites for a given TF are similar, although most often not identical in a DNA sequence. As a result, the binding site motifs are often highly degenerate, which brings in some challenges to build a model for these signals (20). Thus, the computational detection of these cis-regulatory DNA segments within a genome of interest is a major challenge. Furthermore, the relatively short length of binding motifs represented by the PWMs multiplies the challenge because the small amount of information they contain may result in a large number of false-positive predictions in genome-wide searches. This scenario can be compensated by combining the PWMs with some other features such as proximity to TSS (21), chromatin structure (21,22) and proximity to other PWM hits (1,2).

Numerous attempts were made to identify CRMs. However, many of the popular methods need prior knowledge of the TFs involved in the clusters. For example, Wasserman and Fickett developed a model to predict/identify the muscle-specific regulatory modules. They considered the known factors associated with skeletal muscle-specific expression, such as Mef-2, Myf, Sp-1, SRF and Tef (23). Some other methods like DiRE and CREME (24,25) identify the CRMs from a list of co-regulated genes. These methods require prior knowledge of co-regulated genes (relatively small number) from expression data for a given set of genes. The method starts with the preparation of a database of conserved TFBSs for all the TFs from TRANSFAC across the promoter region of human genes and identifying their combinations in a given set of promoters. The method also requires an alternative set of control sequences to evaluate the background distribution of TFBSs and identify the CRMs by statistically evaluating the significant modules.

Nonetheless, these methods do not address one important aspect of CRMs, which is the mutual positioning of the factors composing them, such as a preference for certain distance from each other. As discussed by (26), the relative positioning of the factors is important for understanding the nature of their interactions. In the present article, we propose a new approach where we do not consider *a priori* the set/cluster of factors known to be involved in myogenesis. Instead, we consider all the available factors with respect to statistically significant positional preferences in their mutual positioning with Myogenic differentiation (MyoD). The available methods are helpful in finding regulatory modules from the specific set of genes for a specific biological process. In contrast, our approach is not confined to any individual biological process.

In our approach we take the TF-binding motifs derived experimentally from TRANSFAC database and computationally determined the binding sites on the sequences from the Chromatine immunoprecipitation (ChIP) experiment for specific TF. We also determine the binding site for other TFs in these sequences, and we derive the mutual positioning among the associated factors. Our approach finds the significant association between factors that may reflect their interaction in biological processes. We have also investigated the relationships among the associated factors depending of their distance from the transcription start site and also examined the differences between the functional and similar non-functional binding sites.

Thus, in this work, in addition to identifying the clusters, we analyze the mutual positioning of the factors, i.e. the preferential spacing between them. In this study, we considered all human TFs for which PWMs are available in TRANSFAC database. This enabled us to find the association of muscle specific factors with other non-muscle specific factors. This association may signify the involvement of MyoD with biological processes other than myogenesis and involvement of additional factors in myogenesis.

In this study, we have compared the association of MyoD with other factors in functional binding sites (27) and non-functional MyoD-binding motifs (hits/matches derived from the MyoD unbound sequences) derived as non-overlapping sequences from MyoD bound ChIP-Seq sequences (27). We assume that if certain TFs are significantly over-represented in a close range around binding sites of another factor, such mutual positional preference of the given TFs is related to their common biological function. Combining the computationally searched binding sites with the information concerning association between the factors can also help in determining true binding site of a factor. This way, biologically functional TFBSs can be discriminated from a vast amount of similar yet non-functional motifs: the functional TFBS are more likely to be organized in the CRMs than similar but non-functional motifs.

The results of our work show preferential coupling of the muscle-specific TFBS together with MyoD in the promoter sequences, as well as some novel relationships of MyoD with other non-muscle specific TFs.

## MATERIALS AND METHODS

**Figure 1.**
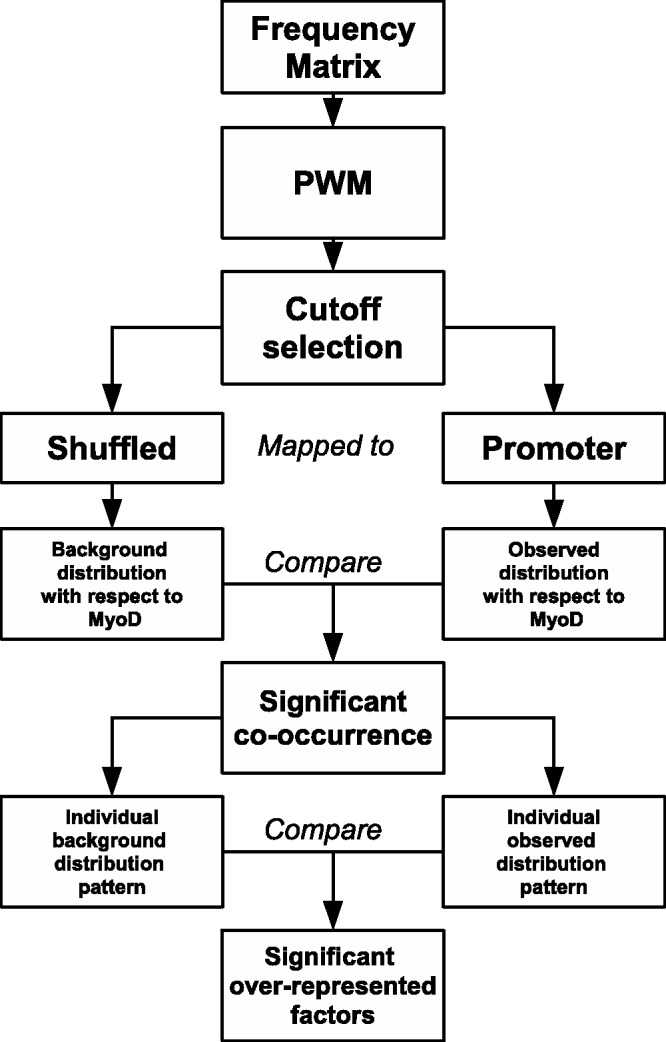
The algorithm to detect the positional association of motifs in close vicinity. The frequency tables are converted into PWM, and the cutoff is determined as described in the text. Each PWM with the corresponding threshold for *OF_r_* = 0.0001 is mapped in both the promoter sequences and shuffled sequences. After comparing the number of occurrences in both the data sets, TFs having significantly higher occurrence in the promoter sequences are selected with the criteria z-score >3. Further, to find out the positional associations of the TFs with respect to MyoD, each observed occurrence distribution is compared with the background distribution, and positions having z-score >10 are selected as preferred positions.

TRANSFAC provides information in the form of base frequency tables for 1226 different TFs. Of these 1226, 721 TFs are found in human. We have adopted base frequency tables for the human TFs from the TRANSFAC database. These tables are often used to find out the binding sites in genomes (28). In our work, we used PWM instead of the frequency tables to map the TFBS in the promoter sequences from human. PWM represents the log-odd probabilities of finding each base at each position in a signal. The whole protocol is outlined in the Figure 1. We implemented the method proposed by Staden (29) to build the PWMs. The background frequencies (30) were calculated from the Database of Transcription Start Sites (DBTSS) (http://dbtss.hgc.jp/) (31) as described in (32,33). The weight for each position of the matrix is derived using the formula described in (32,33), which is a modification of Bucher’s formula (34). Individual weights of the nucleotide corresponding to the matching sequence were summed to calculate the matching score for a sequence (33).

TRANSFAC has three matrices for MyoD (M00001, M00184 and M00929). We have selected the matrix M00184 for our study because this matrix is built from only MyoD sites, and the information content is more than that of M00001. The matrix M00929 is built from E12, E2A, E47, ITF-1; MRF4, Myf-6, MyoD, Myogenin and Tcfe2a-binding sites. The matrix M00929 represents E-box protein rather than MyoD binding site specifically. Furthermore the number of promoters having combination of MyoD BS with E-box BSs for example E12, E2A and myogenin is relatively lower than for the M00184 matrix. For example, E2A + M00184 = 5221 and E2A + M00929 = 2951; E12 + M00929 = 2449 and E12 + M00184 = 3468; myogenin + M00184 = 3599 and myogenin + M00929 = 2951. Considering these facts, we carried out our further analysis with M00184.

### Assigning the threshold

The PWMs calculated with the aforementioned method do not provide us with the threshold score to select the hits from the mapped data. Moreover, we cannot assign a single standard cutoff to all the TFs. Hence, it is essential to determine and assign different specific threshold score to each of the factors. To resolve this problem, we have determined how many sites are likely to arise by chance for any given score for any given TF. To do so, we have created a random promoter data set by shuffling the human promoter sequences using uShuffle while preserving the relative proportion of each nucleotide (35). The DBTSS database was used for the shuffling; therefore, the shuffled sequence database contained sequences of the same number and length. All the 721 factor’s PWMs were mapped with a varying range of threshold to the shuffled sequence data set; thus, these hits tell us how many hits may be obtained by chance for each threshold.

We have determined a specific threshold for each PWM by estimating the number of false positives predicted by the PWM in randomized sequences. To determine a threshold that would result in an acceptable number of the false-positive predictions, we calculated the number of hits for each threshold for each TF based on the shuffled sequences, and we term this as ‘Randomized Occurrence Frequency’ (OF_r_).

We assume that the sites recognized as positive from the randomized sequences are the false positives. We calculate *OF* as the average number of positive predictions per base pair in the random shuffled data set:
(1)
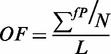

where *fP* is the number of sites predicted in the shuffled sequences by the given PWM, *N* is the total number of sequences in the shuffled sequence database, and *L* is the length of the sequence subtracting the length of the PWM. We will use the notation *OF_r_* to designate occurrence frequency calculated from the shuffled sequence data set. Therefore, the higher the occurrence frequencies from the shuffled sequences are, the lower is the specificity.

Now, we calculate *OF_r_* with the aforementioned formula for each threshold, and to avoid the selection of the false-positive occurrences, we take the *OF_r_* of 0.0001. With this threshold, we would detect minimum level of false positives from the promoter sequences.

We iteratively calculate the *OF_r_* for each cutoff. We start to calculate *OF_r_* for each TF with a high cutoff and check the *OF_r_* after each change in cutoff during the iteration. If the *OF_r_* reaches 0.0001, we stop further decrement of the cutoff for the TF and if *OF_r_* <0.0001, we decrease the cutoff by 0.1 and again calculate *OF_r_*.

Therefore, we assigned the threshold for each factor for selected *OF_r_* of 0.0001, which means average of 1 hit in every 10 000 shuffled sequences at each position. The value of *OF_r_* is empirically selected to restrict the level of false positive predictions by the search procedure.

### Finding the distribution of TFs around the TF of interest

As aforementioned, we have selected the process of ‘myogenesis’ as a study case and have selected to analyze MyoD, as it plays a vital role in the process. We calculated the distribution of factors around the MyoD within the range of ±100 bp. This is because we want to screen the factors that co-occur close to the MyoD inside the given interval. We term these factors as co-occurring with MyoD, i.e. the factors found to occur in combination with MyoD within ±100 bp interval in the proximal promoter region. To determine the distribution of MyoD with itself, we used matrix M00184 and M00929 both for MyoD and calculated the distribution with respect to each other. However, the sites selected in this step do not ensure that they are truly interacting and have biological significance. The distribution of the TFs found around myogenic TF MyoD may be arbitrary.

In addition, we have mapped these PWMs in the MyoD bound ChIP experiment sequences (27). Here, we have incorporated one more constraint, i.e. we have selected the matches only around the center of the MyoD bound sequences (27). The reason of adding the constraint is that in the ChIP-seq bound sequences, MyoD is likely to be bound close to the center of the sequences. Even though while computationally determining the binding sites, we may encounter many hits in the whole sequences, and many of them would be non-functional binding sites. Thus, to avoid the false-binding sites, we have considered only the matches that lie in the central region (up to −20 bp upstream and +20 bp downstream from the center position) on the MyoD bound sequences (27).

### Statistical significance of the co-occurring TFs

To determine that the occurrence of the factors in combination with MyoD is not random, we calculate the statistical significance for each combination of the co-occurring factors. With the threshold obtained for *OF_r_* of 0.0001, we computationally mapped all the 721 PWMs into the shuffled database and find the distribution of other factors around the factor of our interest.

We compared occurrence for each factor around MyoD from the observed distribution with its occurrence in the shuffled sequence database and calculated the z-score with the formula as described in (33). We selected the distribution of the factor for further analyses if that has *z-score* >3.

Same statistical criteria have been implemented to determine the positional preference of the studied factors around MyoD-binding sites in DBTSS. The z-score ≥10 is selected as a cutoff to designate any position as a preferred location of the binding site of factors with respect to MyoD. As in our approach, we do not consider positional bins, the observed and the expected counts are small. Therefore, in addition to the z-score, we have performed the ‘exact binomial test’ to determine the preferred position. The R package is used to calculate the exact binomial *P*-value (36).

## RESULTS

In our study, we used the information from the TRANSFAC database focusing on *de novo* discovery of the CRMs, based on the mutual positioning of TF-binding motifs. We confined our study to the pairwise combinations of the 721 human TFs with a key TF controlling the process of myogenesis, MyoD (37). We mapped the binding sites of MyoD and other factors from TRANSFAC in the human promoter sequences from the DBTSSs (http://dbtss.hgc.jp/) (31) as well as in experimentally identified MyoD-binding sites (27). From these computationally mapped binding sites, we evaluated the distribution of all the factors with respect to their mutual occurrence and distance between them.

### Factors co-occurring with MyoD in human promoters

We have considered all 721 TRANSFAC frequency tables related to human TFs. We calculated PWMs and their respective false-discovery thresholds. These new PWMs were used to search for the similar motifs from 32 042 human promoter sequences (from −1000 to 201 bp around the TSS) and also in the MyoD bound ChIP experiment sequences (27).

We identified the factors having close positional association with MyoD in the range of ±100 bp following earlier studies (see later in the text).

To find the differences from the background distribution, we mapped the same PWMs in the shuffled sequences. Then, we determined how many times each of the factors is found together with MyoD in both the data sets. Wasserman and Fickett (23) found that in case of cis-regulatory elements, most of respective binding sites are positioned within 100 bp from each other. As in our approach, we are looking on the binding sites for only a pair of factors at a time, we considered the distance between them to be at most 100 bp, and therefore, we analyzed only factors found within this interval from MyoD.

A straightforward mechanistic model proposed by Teif *et al.* (38) and based on the experimental study of Drosophila embryonic development by Fakhouri *et al.* (39) explained a possible reason of the preferred distance between BS for a repressor/activator transcription regulation in synthetic enhancers. They proposed a quantitative description of the nucleosome-dependent regulation of the gene expression at short genomic distances. They have showed the preferred distance between the adjacent functional TFBS to be 50–60 bp, which is mediated by nucleosome and TF interactions. Our interval 100 bp covers TF–TF interactions from (37) and also searches for TF–TF interactions in the adjacent area. We also calculated number of TF–TF interactions for various interval lengths to check dependence of the discovered here effects of the interval length.

We calculated the z-score (difference in TF occurrence frequencies in promoter sequences and randomized sequences, in units of standard deviations for the former) for each TF in combination with MyoD and selected those factors that have z-score values above 3. We obtained a large number of TFs with higher z-scores, and the full distribution of the z-scores follows a normal distribution (Supplementary Figure S1).

TF-binding information in TRANSFAC is redundant: some factors are represented by more than one matrix, and certain matrices for different factors are extremely similar. Some of this reflects a biological reality that different proteins can bind related sequences, and some of it is technical and stems from the fact that separate studies have reported independently the binding site preferences of a given factor. Numerous studies have been conducted to address this by comparing and clustering PWMs according to their similarity (40–42). A similar study specifically performed for muscle-specific factors showed that MyoD can be grouped with E47, E12, E2A, myogenin, ABA-responsive element binding factor 6 (AREB6) and Lmo2 based on their matrix similarity (42). According to the previous studies, E-proteins have been shown to dimerize with MyoD to bind to DNA together (27,37). Certainly, in our study, we too found these factors positioned overlapping with MyoD in a significant number of promoters. We therefore should check whether the ‘co-occurrence’ of these TFs with MyoD is real or simply a consequence of redundancy of their binding sites. TFs for which the PWM is similar to that of MyoD (e.g. those for E-proteins and other bHLH factors, see Supplementary Table S1) fall at the same place as MyoD itself. These TFs were removed from the subsequent analyses.

The factors found to have significant co-occurrence with MyoD but are not identical to the MyoD motif pattern are listed in the Tables 1–4. We sorted these factors according to the number of promoters in which they occur together with MyoD (Tables 1–4). Tables 1–3 and Supplementary Table S1 include the factors co-occurring with MyoD in >500 promoters. Factors found co-occurring with MyoD in <500 promoters are reported in Table 4. The number of factors co-occurring with MyoD in >500 promoters and having z-score >3 was found to be 48 of 721, and association of the majority of these factors with MyoD was confirmed to be essential for the process of myogenesis (see later in the text).

**Table 1.** These factors are reported previously to function with MyoD

TF name	TRANSFAC id	No. of promoters with z-score	Consensus from TRANSFAC	Significant positions with *P*-values	
AML1	M00751	903 (34.47)	TGTGGT	−90 (=2.36e-05)	(43)
				−45 (=8.96e-05)	
				−8 (=4.235e-10)	
				8 (=1.084e-06)	
				90 (=4.293e-06)	
AML1a	M00271	903 (34.47)	TGTGGT	−90 (=2.36e-05)	(43)
				−45 (=8.96e-05)	
				−8 (=4.235e-10)	
				8 (=1.084e-06)	
				90 (=4.293e-06)	
TEF-1	M00704	756 (22.82)	GRRATG		(44)
MEF-2	M00233	502 (66.37)	NNTGTTACTAAAAATAGAAMNN	−67 (<2.2e-16)	(45)
				−66 (=0.0007159)	
				−65 (<2.2e-16)	
				−32 (=0.01024)	
				−31 (=7.629e-12)	
				31 (=1.174e-13)	
				32 (=4.35e-06)	
				65 (<2.2e-16)	
				66 (=0.006063)	
				67 (<2.2e-16)	
				68 (=0.0192)	

The table summarizes the top significantly co-occurring factors with MyoD in >500 promoters and are not identical to the MyoD motif pattern. The positions in the last right column are identified to be significant after comparing with the background; only those positions that have z-score above 10 and *P*-value below 0.005 were selected.

**Table 2.** These factors are known to be involved in myogenesis

TF name	TRANSFAC id	No. of promoters with z-score	Consensus from TRANSFAC	Significant positions with *P*-values	
NFAT1	M01281	1123 (44.14)	GGAAAA	−39 (=0.0009199)	(46)
Pitx2	M00482	735 (77.48)	WNTAATCCCAR	−27 (<2.2e-16)	(47)
				−23 (<2.2e-16)	
				−11 (<2.2e-16)	
				10 (<2.2e-16)	
				22 (=7.332e-15)	
				26 (<2.2e-16)	
MAZ	M00649	613 (55.13)	GGGGAGGG	−42 (=0.02665)	(48)
				−34 (=0.02665)	
				29 (=0.006371)	
				41 (=0.01854)	
				44 (=0.01854)	
Meis2	M01488	521 (7.73)	NANNASCTGTCAAWNN	−2 (<2.2e-16)	(49)
				2 (<2.2e-16)	
MEIS1	M00419	521 (12.29)	NNNTGACAGNNN	−3 (<2.2e-16)	(50)
				3 (<2.2e-16)	

The table summarizes the top significantly co-occurring factors with MyoD in >500 promoters and are not identical to the MyoD motif pattern. The positions in the last right column are identified to be significant after comparing with the background; only those positions that have z-score above 10 and *P*-value below 0.005 were selected.

**Table 3.** These factors are not reported earlier to function with MyoD

TF name	TRANSFAC id	No. of promoters with z-score	Consensus from TRANSFAC	Significant positions with *P*-values	Expressed in C2C12
Kid3	M01160	5539 (66.06)	CCACN	−6 (=1.539e-09)	No
				−2 (<2.2e-16)	
				1 (<2.2e-16)	
				2 (<2.2e-16)	
				22 (<2.2e-16)	
ELF1	M01266	1618 (72.90)	AGGAAG	−51 (=0.01598)	Yes
				25 (=0.01047)	
				52 (=0.0008859)	
ZNF333	M01230	1407 (12.61)	ATAAT		No
Ikaros	M01169	1037 (93.70)	KYTGGGAGGN	−36 (<2.2e-16)	Yes
				−34 (=0.1149)	
				−20 (=0.1149)	
				−13 (<2.2e-16)	
				−7 (=0.02247)	
				−13 (<2.2e-16)	
				36 (=7.006e-12)	
Churchill	M00986	978 (5.25)	CGGGNN		No
Lyf-1	M00141	905 (95.84)	TTTGGGAGR	−36 (<2.2e-16)	Yes
				−14 (<2.2e-16)	
				13 (<2.2e-16)	
				35 (<2.2e-16)	
HOXA13	M01292	896 (27.70)	ATAAMA		Yes
E2F	M00803	846 (3.74)	GGCGSG	−47 (=1.35e-12)	Yes
				47 (<2.2e-16)	
MAFB	M01227	843 (19.49)	GNTGAC	−5 (<2.2e-16)	Yes
				5 (<2.2e-16)	
PPARG	M01270	820 (69.35)	AGGTCAN	−84 (=0.02037)	Yes
				−83 (=2.788e-15)	
				−65 (=1.745e-13)	
				−16 (<2.2e-16)	
				−14 (<2.2e-16)	
				−4 (=0.002266)	
				3 (=0.02707)	
				13 (<2.2e-16)	
				15 (<2.2e-16)	
				82 (=9.824e-09)	
				83 (=0.001561)	
T3R	M00963	791 (50.12)	MNTGWCCTN	−83 (=5.434e-08)	No
				−65 (=2.594e-05)	
				−16 (<2.2e-16)	
				−14 (<2.2e-16)	
				−4 (<2.2e-16)	
				3 (<2.2e-16)	
				13 (<2.2e-16)	
				15 (<2.2e-16)	
				82 (=2.593e-08)	
				83 (=0.001544)	
HNF4	M01032	791 (28.14)	AGKYCA	−63 (=4.591e-06)	Yes
				−23 (<2.2e-16)	
				23 (<2.2e-16)	
LEF1	M00805	707 (23.79)	TCAAAG	16 (=1.407e-06)	Yes
ARP-1	M00155	661 (59.75)	TGARCCYTTGAMCCCW	−80 (=3.942e-08)	No
				−67 (=6.71e-13)	
				−18 (<2.2e-16)	
				−16 (=3.942e-08)	
				−6 (=0.02871)	
				16 (=7.885e-05)	
				18 (<2.2e-16)	
				25 (=0.03577)	
				44 (=0.01283)	
				67 (=0.0003216)	
				80 (=1.373e-07)	
				81 (=0.03577)	
				82 (=0.01283)	
PKNOX2	M01411	655 (12.16)	NANSRSCTGTCAATNN	−2 (<2.2e-16)	Yes
				2 (<2.2e-16)	
HOXA4	M00640	612 (60.46)	AWAATTRG	−81 (=0.006691)	
				−80 (=0.006691)	
				−79 (=9.953e-13)	
				−78 (<2.2e-16)	
				−77 (<2.2e-16)	
				−76 (=7.216e-12)	
				−20 (<2.2e-16)	
				20 (<2.2e-16)	
				76 (=1.189e-09)	
				77 (<2.2e-16)	
				78 (<2.2e-16)	
				79 (=1.563e-06)	
				80 (=1.563e-06)	
				81 (=0.0006786)	
ETS2	M01207	611 (54.11)	CTTCCTG	−64 (=0.005913)	Yes
				−42 (=0.01879)	
				−41 (=0.005913)	
				−37 (=0.01879)	
				−34 (=0.01879)	
				−9 (=0.005913)	
				6 (=0.008909)	
				47 (=0.02462)	
				51 (=0.02462)	
PREP1	M01459	542 (12.19)	NRNSASCTGTCAAWNN	−2 (<2.2e-16)	Yes
				2 (<2.2e-16)	
TBX5	M01044	541 (30.82)	CTCACACCTT	−35 (<2.2e-16)	
				−14 (=3.398e-09)	
				−2 (<2.2e-16)	
				2 (<2.2e-16)	
				14 (=1.087e-06)	
				35 (<2.2e-16)	
CKROX	M01175	534 (41.87)	SCCCTCCCC	41 (=0.001077)	Yes
PU.1	M00658	526 (38.59)	WGAGGAAG	76 (=0.002891)	
				83 (=3.386e-05)	
				99 (=0.002891)	
SREBP-1	M00220	515 (47.42)	NATCACGTGAY	−90 (=1.377e-05)	Yes
				−58 (<2.2e-16)	
				−9 (<2.2e-16)	
				8 (<2.2e-16)	
				57 (=6.051e-07)	
				89 (=1.595e-08)	
				90 (=0.001458)	
Sp1	M00933	510 (38.68)	CCCCGCCCCN		Yes
Pax-4	M00377	508 (56.64)	NAAWAATTANS	−80 (=8.752e-05)	No
				−79 (=9.388e-16)	
				−78 (<2.2e-16)	
				−77 (=6.032e-12)	
				−76 (=4.789e-11)	
				−21 (<2.2e-16)	
				20 (<2.2e-16)	
				57 (=0.03888)	
				74 (=0.01283)	
				75 (=3.045e-07)	
				76 (=1.632e-11)	
				77 (<2.2e-16)	
				78 (=9.832e-06)	
				79 (=9.832e-06)	
				80 (=0.0009783)	

The table summarizes the top significantly co-occurring factors with MyoD in >500 promoters and are not identical to the MyoD motif pattern. The positions in the fifth column are identified to be significant after comparing with the background; only those positions that have z-score above 10 and *P*-value below 0.005 were selected.

**Table 4.** The table summarizes the factors, other than 48 in Tables 1, 2 and 3, whose co-occurrence with MyoD found to be significant (z-score > 3) in <500 promoters

TF name	TRANSFAC id	No. of promoters with z-score	Consensus from TRANSFAC	Significant positions with *P*-values
M00499	STAT5A	497 (23.77)	NNNTTCYN	
M00444	VDR	496 (39.30)	GGGKNARNRRGGWSA	−9 (=0.002345)
				0 (=8.252e-05)
				8 (=0.0003963)
				11 (=0.001719)
				33 (=0.001719)
M00231	MEF-2	492 (55.18)	NNNNNNKCTAWAAATAGMNNNN	−67 (<2.2e-16)
				−66 (=0.02747)
				−65 (<2.2e-16)
				−32 (=0.008367)
				−31 (=4.207e-13)
				31 (=9.454e-13)
				32 (=0.005949)
				65 (<2.2e-16)
				66 (=0.001677)
				67 (<2.2e-16)
				68 (=0.0189)
M01181	Nkx3-2	485 (12.25)	TRAGTG	
M00983	MAF	480 (42.49)	NGCTGAGTCAN	−44 (=0.006943)
				−32 (<2.2e-16)
				−6 (<2.2e-16)
				5 (<2.2e-16)
				31 (<2.2e-16)
				43 (=0.001297)
M01177	SREBP2	467 (19.24)	NNGYCACNNSMN	−1 (<2.2e-16)
				1 (<2.2e-16)
M00706	TFII-I	461 (36.67)	RGAGGKAGG	
M00971	Ets	459 (34.81)	ACTTCCTS	6 (=0.002202)
M00418	TGIF	457 (11.06)	AGCTGTCANNA	−4 (<2.2e-16)
				3 (<2.2e-16)
M01275	IPF1	453 (9.20)	CATTAR	21 (=7.575e-09)
M00148	SRY	441 (32.54)	AAACWAM	
M01395	MRG2	440 (7.66)	NANNASCTGTCAANNN	−2 (<2.2e-16)
				2 (<2.2e-16)
M00695	ETF	438 (10.38)	GVGGMGG	
M00083	MZF1	432 (30.24)	NGNGGGGA	−3 (<2.2e-16)
				3 (<2.2e-16)
M00979	Pax-6	429 (37.31)	CTGACCTGGAACTM	−75 (=0.001308)
				−72 (=5.749e-05)
				−26 (<2.2e-16)
				−24 (=0.0002887)
				−7 (=1.733e-06)
				24 (=0.002023)
				26 (<2.2e-16)
				72 (=0.0004781)
				75 (=0.000102)
M01036	COUPTF	422 (27.57)	NNNNNTGACCYTTGNMCNYNGMN	−79 (=6.733e-05)
				−8 (<2.2e-16)
				7 (=6.733e-05)
M00339	c-Ets-1	421 (36.77)	RCAGGAAGTGNNTNS	3 (=5.815e-05)
M01153	PXR	420 (30.02)	NNAGTTCA	−71 (=4.888e-06)
				−22 (<2.2e-16)
				−20 (=0.0006013)
				20 (=0.0001583)
				22 (<2.2e-16)
				71 (=4.855e-06)
				76 (=2.906e-05)
				77 (=0.0007775)
M00175	AP-4	420 (3.28)	VDCAGCTGNN	−16 (=7.462e-08)
				0 (<2.2e-16)
M00974	SMAD	419 (22.43)	TNGNCAGACWN	−36 (=5.562e-07)
				−6 (<2.2e-16)
				5 (<2.2e-16)
M01273	SP4	405 (35.67)	SCCCCGCCCCS	
M00483	ATF6	400 (5.84)	TGACGTGG	
M01247	Nanog	393 (41.12)	NNWNNANAACAAWRGNNNNN	−80 (=0.009703)
				−75 (=0.009703)
				−71 (=0.002352)
				−24 (=0.009703)
				32 (=0.006446)
				74 (=0.00165)
M00468	AP-2rep	391 (26.36)	CAGTGGG	
M00257	RREB-1	388 (34.44)	CCCCAAACMMCCCC	
M00646	LF-A1	388 (19.73)	GGGSTCWR	
M01066	BLIMP1	385 (38.04)	AGRAAGKGAAAGKR	
M01248	Dax1	384 (26.12)	NNRNNNNAAGGTCANNNNNN	−12 (=4.454e-07)
				−5 (=5.986e-08)
				5 (=2.853e-08)
				12 (=1.371e-06)
M00982	KROX	377 (28.43)	CCCGCCCCCRCCCC	
M01200	CTCF	375 (21.32)	NNNGCCASCAGRKGGCRSNN	−1 (<2.2e-16)
				1 (<2.2e-16)
M00480	LUN-1	364 (30.84)	TCCCAGCTACTTTGGGA	−20 (=0.001019)
				−19 (<2.2e-16)
				18 (<2.2e-16)
				19 (=0.0003236)
M01252	E2F6	363 (18.70)	CNTTTCNT	
M00793	YY1	355 (28.46)	GCCATNTTN	−93 (=7.798e-05)
				−44 (<2.2e-16)
				−7 (=0.002028)
				43 (<2.2e-16)
M00264	Staf	346 (30.35)	MNTTCCCAKMATKCMWNGCRA	−90 (=1.187e-09)
				−9 (=1.658e-11)
				8 (=5.959e-13)
				88 (=0.002063)
				89 (=0.002063)
M01269	NURR1	344 (22.12)	YRRCCTT	−5 (=1.256e-13)
				4 (=4.731e-07)
M00794	TTF-1	341 (20.71)	NNNNCAAGNRNN	−51 (=0.0001002)
				−10 (<2.2e-16)
				10 (<2.2e-16)
M00733	SMAD4	337 (20.70)	GKSRKKCAGMCANCY	−6 (<2.2e-16)
				5 (<2.2e-16)
M00972	IRF	336 (37.89)	RAAANTGAAAN	16 (=0.009956)
				53 (=0.002033)
				63 (=0.009956)
M01214	ESE1	336 (34.39)	DRYTTCCTGW	−89 (=0.004255)
				−6 (=0.0008684)
				6 (=0.0006748)
				9 (=2.403e-05)
				45 (=0.003028)
M01168	SREBP	333 (26.12)	NNNNYCACNCCANNN	−58 (=0.0001974)
				6 (=1.218e-05)
				90 (=0.0005096)
M01217	NUR77	326 (26.40)	NTGACCTTBN	−99 (=0.0006919)
				−12 (=3.477e-11)
				−5 (=3.18e-07)
				12 (=3.141e-06)
M01342	CDP	321 (28.71)	ACCGNTTGATYANSWNN	−54 (=2.251e-05)
				−5 (<2.2e-16)
				4 (<2.2e-16)
M01295	ATF5	319 (27.00)	CYTCTYCCTTA	
M00746	Elf-1	316 (28.13)	RNWMBAGGAART	
M00532	RP58	312 (14.67)	NNAACATCTGGA	−1 (<2.2e-16)
				1 (<2.2e-16)
M00965	LXR	309 (22.62)	YGAMCTNNASTRACCYN	−59 (=1.589e-05)
				−10 (<2.2e-16)
				9 (<2.2e-16)
M00762	PPAR	308 (22.07)	RGGNCAAAGGTCA	−8 (=4.627e-06)
				7 (=0.0001904)
M01028	NRSF	308 (21.64)	GYRCTGTCCRYGGTGCTGA	−10 (=3.38e-08)
M00721	CACCC-binding	307 (28.01)	CANCCNNWGGGTGDGG	−84 (=1.736e-05)
				2 (=0.00149)
				89 (=0.0002928)
M00665	Sp3	305 (21.98)	ASMCTTGGGSRGGG	
M00650	MTF-1	305 (20.10)	TBTGCACHCGGCCC	−49 (=1.716e-14)
				0 (<2.2e-16)
				49 (=1.844e-11)
M00726	USF2	300 (7.25)	CASGYG	

The positions in the last column are identified as in Tables 1, 2 and 3.

**Figure 2.**
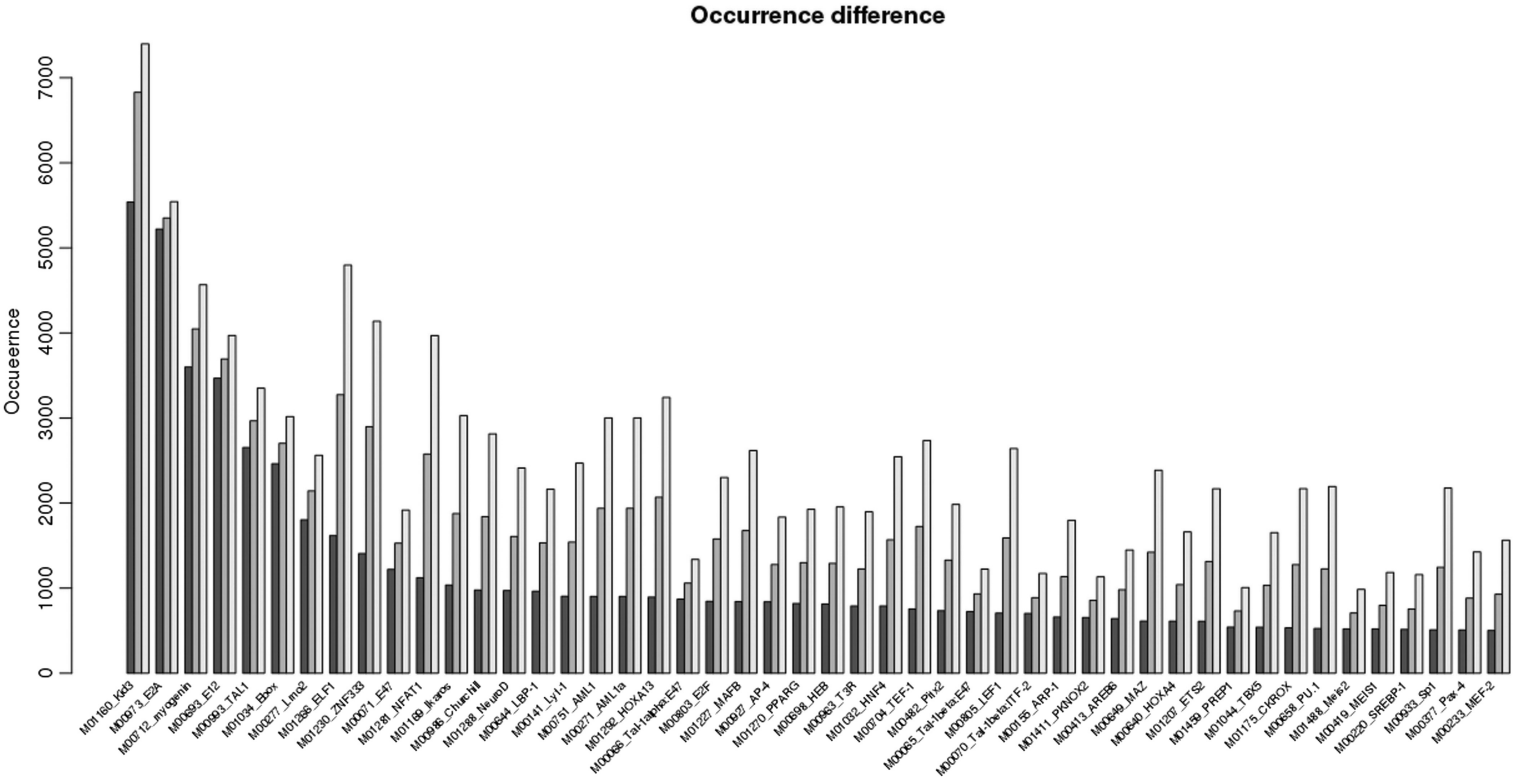
Distribution of factors around MyoD. The variation in occurrence of 48 factors in combination with MyoD in varied window. Figure shows the differences in occurrence of 48 factors in combination with MyoD in varied window size 200 bp (black bar), 500 bp (dark gray bar) and 1000 bp (light gray bar). The occurrences are shown in the *y*-axis. These 48 factor’s occurrences are found to be significant (z-score >3 comparing with a background), and they are found in >500 promoters when analyzed with window size 100 bp.

We have investigated the dependence of the number of MyoD-TF pairs on the length of the region. The result is demonstrated in Figure 2. The figure shows the difference in the number of hits when the distance range is changed to 200 bp (black bar), 500 bp (dark gray bar) and 1000 bp (light gray bar) keeping MyoD-binding site at the center for the factors selected from the Tables 1–3 and Supplementary Table S1. From the figure, we can see that the differences in the number of occurrences vary for the particular factors in different distance ranges. After investigating the cause of such variations, we found that the length of the motifs seems to be a primary factor. For example, when the range is increased from 200 to 1000 bp, the number of hits/occurrences for Kid3 and ZNF333 also increased. This is expected, as the co-occurrence of Kid3 and ZNF333 with MyoD is relatively high because of the short motif length. However, for factor E2A, the difference of number of occurrences is small, which means that it is less likely that we would get high number of occurrences if the range is increased. The motif of the factor E2A is similar to that of the MyoD. This kind of distribution is similar with other E-box proteins as well. The lesser number of hits of these factors after increasing the distance/range implies that MyoD like E-box motifs are locally concentrated around the MyoD. A lesser increase in the occurrence of these E-box factors binding sites (similar to that of MyoD BS) can also be a consequence of the fact that the MyoD binds at the E-box binding sites as described by Tapscott (27).

For the factor Meis, the difference in the occurrence number is less with the increased range, which is expected, as the factor is functionally associated with MyoD and hence may be also closely situated on the DNA sequence. However, the distribution/occurrence of the other factors known to be involved in the process of myogenesis, like AML1 and MAZ rises significantly with the increase in range of distance from MyoD-binding sites, which is unexpected. For factors such as PKNOX and PREP1, the number of occurrences does not increase with the increase of the range. This indicates that PKNOX and PREP1 BSs prefers to co-localize with MyoD BS these factors were not reported to be associated with the myogenesis or to function with MyoD. However, this observation indicates that the localized distribution of the BSs similar to that of MyoD BS may explain the fact that MyoD or MyoD like BS are not distributed in wider genomic regions, rather they are concentrated at certain regions of the genome.

Among the top 48 factors listed in the Tables 1–3 and Supplementary Table S1, 23 are reported in previous studies to have some activity in muscle development or some interactions with MyoD or are associated together in the promoter area of genes. For example, MyoD activates the mouse MafB promoters (51). E-protein HEB is one of the primary E-proteins to regulate skeletal muscle differentiation as per the findings of (52). Recently, the sequential association between MyoD, myogenin, Myf5 and HEB has been established by (53). TEF-1 from the family of TEAD TFs (54) was found to regulate tissue-specific gene expression in muscle and placenta (55,56). Pitx2 is an upstream activator of extraocular myogenesis and survival (57).

Apart from the factors previously determined to function with MyoD, we observed the association of MyoD with other factors not specific to muscle or involved with myogenesis co-occurring with MyoD in a significant number of promoters, e.g. PREP1, NFAT1, Ikaros, Lyf-1, Sterol regulatory element binding proteins (SREBP), AREB6 and Pax4. Though not well established, some indication of association of some of these factors with MyoD in some biological process can be found in previous literature. For instance, (58) indicated the co-occurrence of MyoD and Ikaros in the proximal 1.5 kb region of genes encoding melanin-concentrating hormone receptor. A previous study by (59) has showed that activation and over-expression of PPARg promote adipogenic conversion of myoblasts. The functional interaction between MyoD and T3R in regulation of avian myoblast differentiation is shown in (60). Krox-like binding sites along with MyoD-like binding sites are present in myoblast-specific domain of muscle-specific enhancer (61). Deletion and site-directed mutation experiments demonstrated that at least 2 Krox-like sequences are required for enhancer activity in myoblasts (62). Other works (27,63–65) also showed that MyoD binding overlaps with various other TFs, although the overlap is not systematic: the binding sites of some TFs such as E2F, SRF or NRSF tend not to co-occur with those of MyoD (Supplementary Table S2).

Though NFAT belongs to the family of nuclear factors of activated T-cells, we found this factor to have the significant preference to locate at 39 bp upstream from MyoD. NFAT signaling is required for primary myogenesis by transcriptional cooperation with MyoD (66). Involvement of MyoD in glucose metabolism has been reviewed by (67). They indicated other factors involved in this mechanism along with MyoD such as MEF2A, SREBP, C/EBP and NF-1 in insulin-mediated GLUT4 gene expression, which belongs to the glucose transport family that is expressed in the muscle adipose tissue and heart. In our study too, we found that these factors have a significant specific spacing with regards to the MyoD-binding site in a large number of promoters. The substantial occurrence of the binding sites of these factors within a close proximity around MyoD may have biological significance.

**Figure 3.**
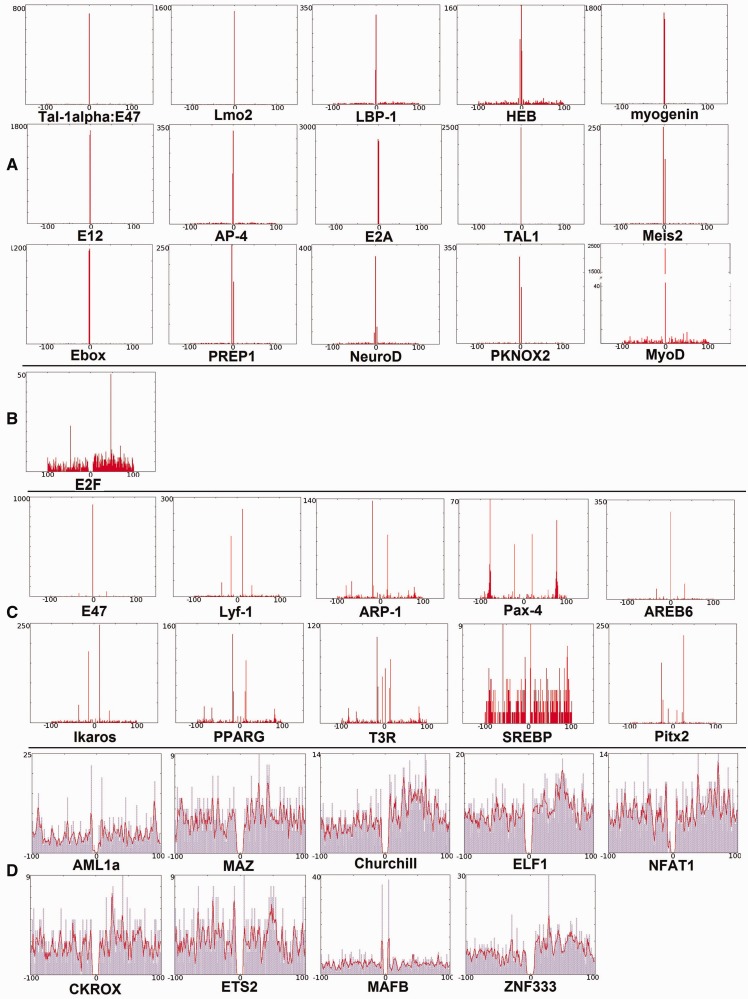
Comparison of distributions of individual factors with background around MyoD. Each panel shows the actual distribution of an individual factor around MyoD in each position within the range of ±100 bp. The factors selected here found to co-occur in >500 promoters. Depending on the positional distribution of these factors, they are divided into four groups and represented as (A) factors with binding motifs highly overlapping with MyoD; (B) factors with single occurrence peak apart from MyoD; (C) factors with several distinct peaks upstream and downstream of MyoD and (D) factors broadly distributed upstream and downstream of MyoD. The *x*-axis represents both upstream and downstream distance from MyoD positioned at ‘0’. The *y*-axis represents the number of promoters found to have the aforementioned factors in combination with MyoD. The blue plot in D represents the occurrence at individual positions, and the red plot represents the running average of 3 of the individual occurrence at each position from DBTSS promoter database.

Provided that the MyoD BSs are GC-rich, it is more likely that we would obtain more MyoD BSs in GC-rich regions than elsewhere. It is also possible that we would discover there the enrichment of other GC-rich TFBS co-occurring with MyoD BSs. This is also reflected in the factors in Tables 1–4. To determine whether the association of the factors is contributed by the GC-content biases, we have partitioned the DBTSS promoters into CpG island containing (CpG+) and non-CpG island containing (CpG−) promoters using program Promoter Classifier (68). In these partitioned promoter sequences, we have analyzed the co-occurrence of the TFs with MyoD. The result is presented in the Supplementary Table S3. From the Table it is clear that the proportion of the co-occurring factors in the whole DBTSS database and the partitioned database are similar. For example, proportion of co-occurrence of myogenin with MyoD is found to be 0.12565 (promoters found to have MyoD-binding sites in the DBTSS database divided by total number of promoters in the database) in CpG+ promoters and 0.11407 in CpG− and 0.11231 in complete DBTSS. Though we can see the slightly higher proportion in CpG+ as compared with the CpG− promoters, which may be contributed by the GC-rich promoter effect, overall similar proportions show that promoter’s GC content is not significantly affecting the co-occurrence of these factors. However, the GC content might be responsible for the distribution pattern of the factors having relatively short motif length (Figure 3D), where the factors are almost evenly distributed around MyoD and do not have any preferred location with respect to the position of MyoD.

### Mutual positioning of factors with respect to MyoD

In addition to the screening of the factors co-occurring with MyoD at some average distance, we investigated the individual distribution of each factor around MyoD. In this analysis, we again aligned promoters with respect to MyoD and calculated the actual occurrence distribution of each factor separately for all factors listed in the Tables 1–3 and Supplementary Table S1. In all promoters, we calculated the number of occurrences of these TFs within ±100 bp of MyoD-binding site and used our false-discovery estimation method to calculate a z-score for each TF to determine whether the distributions are significantly different.

The factors found to be overlapping with MyoD in the previous section have similar observed and background distributions but are remarkably over-represented in the DBTSS promoter sequences (Figure 3A). The figure also shows the complete distribution of MyoD with itself. However, AREB6 and E47 despite having similar peak in the overlapping area also have another peak upstream and downstream (Figure 3C). These factors (AREB6 and E47) have a number of occurrences similar to the background in the overlapping area; yet, they rather have outnumbered the background occurrence in further upstream and downstream areas. The peak in the overlapping area may be caused by the similar motif pattern between MyoD and AREB6 and E47; however, the peaks in the upstream and downstream positions might indicate the preferred positioning of these factors with respect to the MyoD.

### Positional preferences of the factors with respect to MyoD

From this analysis, we have determined the significant positional preference of occurrence of each factor with respect to MyoD. Depending on the calculated z-score, we selected those positions that have a z-score above 10 and *P*-value <0.005. These positions are highly significant and are represented in the column 5 of Tables 1–4.

We observed that some factors show remarkable differences in the distribution and show distinct positional preference. The positional preference can be seen where a factor at a specific distance from MyoD is found in a large number of promoters. Among the 48 factors selected in the previous section with *P* < 0.005, 43 show preferences (listed in Tables 1–3 and Supplementary Table S1). In all, 17 of 43 show the overlapping positional preferences. For the majority of these factors overlapping co-occurrence is confirmed in previous studies except PREP1, PKNOX2, AP-4, LBP-1 and Lmo2. However, some of the factors did not exhibit such significant positional preference, and these factors are found to be evenly distributed around MyoD-binding motifs. The preferred locations for factors like Meis, NFAT1, E-box proteins are found to be precise, whereas no preferred locations for factors like AML1a, MEF-2 are found in close proximity of MyoD. These factors are scattered around MyoD-binding sites.

Other factors found to co-occur significantly in promoters with MyoD but lacking any preferential positions with z-score ≤10 are listed in Supplementary Table S4. Among them, some are found to have biological association with MyoD. CCCTC-binding factor (CTCF) is found co-occurring with MyoD, and the association is recently described by (69). They found that CTCF enhanced myogenic differentiation by directly interacting with myogenic regulatory factors like MyoD and myogenin.

### Patterns of distribution of particular factors around MyoD

From the individual distribution of each factor at each position in the range of ±100 bp around MyoD, we can observe some differences in the distribution patterns. Occurrence of some of the factors is found to be higher in surrounding regions besides the overlapping region. This is expected, as they have no similarity in their motif pattern but are present in abundance upstream and downstream of the MyoD. For example, AML1a, which shows some preferential distance from MyoD at ±90 bp, is highly represented in upstream and downstream areas (Figure 3D). This result might indicate that MyoD has a higher affinity for sequences where AML1a sites are abundant and possibly where AML1 (Runx1) is bound. This would not be fortuitous, as this protein has been shown to bind directly to MyoD in myoblasts (43). Other factors exhibiting this kind of distribution are ELF1, TTF-1, MAZ, MEF-2, Lyf-1, p300, MZF1, ZID, Pax-6, KROX and Nanog. Thus, in addition to the preference for flanking E-box sequences as suggested by (27), MyoD may also prefer binding to the sequence/location enriched with these binding sites.

From these observations, four distinct groups of TFs depending on their positional distribution with respect to MyoD can be seen. Group 1: factors found to highly overlap with the MyoD. Binding motifs of factors in this group closely resemble MyoD; therefore, they have a single peak overlapping with MyoD, and their discovery may be trivial. Representatives of this group are often E-proteins or other classes of bHLH factors: E2A, Myogenin, E12, TAL1, Ebox, Lmo2, NeuroD, LBP-1, Tal-1alpha, E47, AP-4, HEB, PKNOX, PREP1 and Meis2 (Figure 3A). Group 2: factors with single occurrence peak apart from MyoD like E2F (Figure 3B). Group 3: factors with several distinct peaks upstream and downstream of MyoD (Figure 3C), for example: Ikaros, Lyf-1, PPARG, T3R, Pitx2, ARP-1, AREB6, SREBP and Pax-4. Some of the factors in this group are zinc-finger proteins and largely take part in organ development, morphogenesis and also in metabolism. Group 4: factors in this group are broadly distributed upstream and downstream of MyoD with significant representation of zinc-finger proteins in this group. Factors in this group are ELF1, ZNF333, NFAT1, Churchill, AML1, MAFB, MAZ, ETS2 and CKROX. These factors are largely involved in transcription regulation and immune system. Many of these factors, though not all of them, are also found to have GC-rich motifs. There is no particular or distinct preferred position for these factors to be found upstream or downstream (Figure 3D). The abundance of occurrence of GC-rich motifs in the DBTSS human promoters is expected as 72% of the human genome promoters are GC-rich (70). Even as observed in Figure 2, the occurrence of these factors in varied window length (200, 500 and 1000 bp) increases, which implies their general abundance in the promoters. However, the occurrence of the factors of Groups 1 and 3 does not increase for the larger interval length (Figure 2).

### Expression of associated factors in muscle tissue

From our analysis, we have found that some of the non-muscle specific factors co-occur with MyoD in significant number of promoters (Table 3). Now the obvious question would be if these factors are at all expressed in the muscle cell environment. To determine this, we have checked the expression profile of these factors in the previously published expression microarray data (71) from a time course of C2C12 mouse myoblast differentiation. The result is summarized in the Table 3. The last column in the table is marked as ‘Yes’ if we detect any expression in the C2C12 cells and ‘No’ otherwise. From these data, we could see that many (∼60%) of the novel factors found in our study is detectably expressed in the muscle cell environment. This may imply that these binding sites that are in close proximity to MyoD have some important biological meaning yet to be identified. They also may function with MyoD in the process of myogenesis. The other factors with no detectable expression in C2C12 cells have no significant biological importance in the muscle cell environment.

### Association of factors with MyoD in ChIP-seq experiments

Recently, Cao *et al.* (27) used ChIP-sequencing to identify genome wide binding sites of MyoD in mouse muscle cells. MyoD targets in undifferentiated myoblast and in differentiated myotubes were reported. To take advantage of these binding sites and to validate the findings of our study, we have mapped all the PWMs specified for human from TRANSFAC used in our analysis, in these MyoD-bound sequences, with the same constraints like the cutoff and the survey region ±100 bp around the site of MyoD binding.

### Similarity in preferences for factors association in myoblast and myotubes

The distribution of MyoD with factors other than E-proteins is similar in both the surveyed data sets (promoters from DBTSS and the ChIP-Seq bound sequences). Factors primarily mentioned by (27) like AP1, Meis and Sp1 are also found with MyoD in a large number of promoters in both these data sets. Table 5 lists the factors associated with MyoD in large number of promoters in both myotubes and myoblasts. However, the number of hits in the myotubes is significantly higher than that of myoblast sequences in most of the cases except few factors like AP-1 and FRA1 (Table 5). This suggests that more genes might be activated by MyoD in association with these factors (Table 5) during or after differentiation. This can be seen in the fifth column of the table.

**Table 5.** The table represents the factors which show over-representation in ChIP-seq MyoD-bound myoblast or myotube sequences (27)

TRANSFAC IDs	Names	No. of promoters with z-score		z-score
		Myoblast	Myotubes	
M00174	AP-1	376 (70.20)	290 (51.77)	7.70
M01267	FRA1	379 (72.46)	298 (53.34)	7.33
M01034	Ebox		497 (5.08)	
M01207	ETS2	301 (57.97)	327 (54.22)	0.56
M01281	NFAT1	336 (24.96)	367 (24.30)	0.49
M01266	ELF1	710 (54.50)	788 (57.71)	0.26
M01034	Ebox		497 (5.08)	
M00805	LEF1	303 (23.40)	347 (24.92)	−0.41
M00751	AML1	572 (45.26)	652 (55.20)	−0.44
M00971	Ets	252 (38.12)	294 (40.83)	−0.69
M00658	PU.1	273 (34.52)	319 (48.88)	−0.74
M01032	HNF4	267 (19.97)	313 (23.74)	−0.79
M01488	Meis2	370 (21.56)	442 (23.84)	−1.31
M00704	TEF-1	358 (22.47)	437 (31.18)	−1.72
M01395	MRG2	316 (20.59)	388 (25.25)	−1.73
M00277	Lmo2	601 (12.84)	724 (15.37)	−1.89
M00070	Tal-1beta:ITF-2	351 (11.58)	439 (18.67)	−2.19
M00065	Tal-1beta:E47	304 (7.44)	390 (11.00)	−2.51
M01230	ZNF333	326 (3.08)	417 (8.34)	−2.54
M01459	PREP1	346 (18.78)	441 (27.28)	−2.55
M00419	MEIS1	316 (20.79)	412 (30.95)	−2.86
M00066	Tal-1alpha:E47	377 (9.69)	487 (13.29)	−2.93
M01411	PKNOX2	447 (25.08)	572 (32.94)	−2.98
M01346	TGIF1	371 (26.87)	482 (34.22)	−3.03
M00414	AREB6	286 (8.00)	391 (12.10)	−3.57
M01160	Kid3	1428 (19.87)	1750 (30.50)	−3.60
M00698	HEB	590 (38.93)	762 (50.72)	−3.67
M01139	LMAF	233 (34.82)	328 (47.04)	−3.70
M00974	SMAD	221 (26.68)	320 (46.52)	−4.05
M00993	TAL1	767 (14.44)	1004 (26.42)	−4.57
M00644	LBP-1	1170 (89.50)	1488 (116.45)	−4.60
M00071	E47	561 (17.99)	756 (27.68)	−4.64
M00712	myogenin	1331 (31.05)	1683 (48.57)	−4.69
M00176	AP-4	631 (42.93)	855 (63.26)	−5.07
M01227	MAFB	442 (24.17)	628 (37.19)	−5.30
M01288	NeuroD	1037 (74.42)	1380 (105.26)	−5.88
M00693	E12	907 (8.04)	1222 (18.56)	−5.90
M00973	E2A	1087 (5.87)	1446 (17.51)	−6.01

The factors having high number of occurrence in myoblasts and myotubes are selected in this table. The z-score is calculated for the occurrence of these selected factors with MyoD in myoblasts versus myotubes.

As observed in the previous analysis, here too, we found the preferred association of MyoD with E-boxes. However, we also detected preferences for some of the factors other than E-box proteins that were not reported to function together with MyoD in the process of myogenesis in the previous studies. These factors are shown in the Table 6. We have also detected the preferred mutual position of these factors with respect to MyoD in both myoblasts and myotubes. The columns six and seven represent the preferred significant positions in myoblasts and myotubes, respectively. These factors are also found in large number of promoters associated with MyoD in these sequences. Some of the factors found in our previous analysis are also detected here, e.g. PKNOX, TGIF, MAFB, TBX5.

**Table 6.** The table represents the factors that show distinct preferred position with respect to the MyoD in the ChIP-seq MyoD bound myoblast and myotube sequences (27)

TransfacID	Name	No. of promoter in		Pattern	Significant positions in with *P*-values	
		Myoblast	Myotubes		Myoblast	Myotubes
M01411	PKNOX2	447	572	NANSRSCTGTCAATNN	−2 (<2.2e-16)	−2 (<2.2e-16)
					2 (<2.2e-16)	2 (<2.2e-16)
M01227	MAFB	442	628	GNTGAC	−5 (=2.154e-13)	−5 (<2.2e-16)
					5 (=3.565e-14)	5 (=6.504e-16)
M00418	TGIF	397	504	AGCTGTCANNA	−4 (<2.2e-16)	−4 (<2.2e-16)
					3 (<2.2e-16)	3 (<2.2e-16)
M00037	NF-E2	295	282	TGCTGAGTCAY	−55 (=0.02176)	−55 (=0.006288)
					−48 (=0.004337)	6 (=0.007498)
					−43 (=0.02176)	19 (=0.001428)
					−38 (=0.02176)	22 (=0.03312)
					−29 (=0.02176)	24 (=0.03312)
					−19 (=0.004337)	38 (=0.007498)
					−15 (=0.004337)	
					−7 (=1.479e−06)	
					35 (=0.04379)	
					46 (=0.04379)	
					51 (=0.01301)	
M00771	Ets	243	252	ANNCACTTCCTG	−4 (=1.142e-05)	−92 (=0.01814)
					4 (=1.98e-06)	−88 (=0.01814)
					52 (=0.02049)	−58 (=0.01814)
					57 (=0.02049)	−39 (=0.01814)
						−4 (=8.754e-06)
						84 (=0.0001799)
M01139	LMAF	233	328	GSTCAGCAG	−12 (=0.001757)	−5 (<2.2e-16)
					−5 (<2.2e-16)	4 (<2.2e-16)
					4 (<2.2e-16)	6 (=0.02716)
						9 (=0.005792)
						12 (=0.02716)
						31 (=0.02716)
						34 (=0.02716)
						45 (=0.005792)
						47 (=0.005792)
M00531	NERF1a	229	235	YRNCAGGAAGYRNSTBDS	−4 (<2.2e-16)	−70 (=0.000412)
					4 (=2.928e-14)	−58 (=0.0153)
					17 (=0.002413)	−42 (=0.0153)
					20 (=0.01828)	−4 (<2.2e-16)
					27 (=0.01828)	4 (=2.529e-07)
					43 (=0.01828)	16 (=0.01434)
					52 (=0.0002561)	
					61 (=0.01828)	
					70 (=0.01828)	
M00974	SMAD	221	320	TNGNCAGACWN	−6 (=2.069e-11)	−19 (=0.01695)
					5 (<2.2e-16)	−9 (=0.0007924)
						−6 (<2.2e-16)
						5 (=3.374e-14)
						34 (=0.03114)
M01200	CTCF	201	260	NNNGCCASCAGRKGGCRSNN	−1 (=1.68e-12)	−1 (=1.392e-14)
					1 (=9.061e-11)	1 (=2.755e-16)
						54 (=0.002819)
M00701	SMAD3	199	252	TGTCTGTCT	−6 (=0.003092)	−60 (=0.02766)
					5 (=9.911e-09)	−43 (=0.02766)
						−9 (=0.02766)
						−6 (=2.233e-05)
						5 (=2.463e-08)
						18 (=0.0136)
						86 (=0.0136)
M00733	SMAD4	197	258	GKSRKKCAGMCANCY	−39 (=0.003734)	−6 (<2.2e-16)
					−6 (<2.2e-16)	5 (<2.2e-16)
					5 (<2.2e-16)	
M00256	NRSF	144	187	TTCAGCACCACGGACAGMGCC	−10	−91 (=0.0008942)
					(=0.0002676)	−10 (=0.0001039)
					−7 (=1.705e-11)	−7 (=6.921e-08)
					6 (=3.432e-05)	6 (=2.692e-09)
					9 (=0.002057)	9 (=2.353e-05)
M01044	TBX5	138	205	CTCACACCTT	−2 (=2.546e-10)	−2 (<2.2e-16)
					2 (<2.2e-16)	2 (<2.2e-16)
M01028	NRSF	138	183	GYRCTGTCCRYGGTGCTGA	6 (=3.885e-05)	−7 (=3.829e-05)
						6 (=1.942e-11)
M01109	SZF1-1	119	142	CCAGGGTAWCAGCNG	−8 (=0.0005073)	−8 (=0.0005957)
					−5 (=2.005e-06)	−5 (=4.477e-07)
					4 (=4.238e-16)	4 (=8.917e-08)
M01105	ZBRK1	114	146	GGGSMGCAGNNNTTT	−2 (<2.2e-16)	−2 (<2.2e-16)
					1 (<2.2e-16)	1 (<2.2e-16)
					35 (=0.002377)	35 (=0.001127)
M00069	YY1	97	141	NNNCGGCCATCTTGNCTSNW	7 (=0.0002103)	−61 (=0.008413)
						−55 (=0.008413)
						−7 (=0.008413)
						0 (=8.699e-06)
						7 (=0.00348)
M01019	TBX5	78	88	NNAGGTGTNANN	2 (=6.335e-11)	2 (=3.998e-12)
M00960	PR	128	181	NWNAGRACAN	−5 (=5.756e-13)	−5 (<2.2e-16)
					5 (=0.0001041)	5 (=1.599e-11)
						58 (=0.006323)

The table excludes the E-box proteins. The last two columns show the preferred positions in myoblasts and myotubes.

### Differences in preferences for factors association in proximal promoters and enhancers

We used the ChIP-Seq data (27), along with the mouse genome annotation, to identify MyoD-binding sites (from the myoblasts and myotubes data sets combined) that lie within proximal promoters (from 1 kb upstream to 0.2 kb downstream of the TSS), and within distal promoters (from 10 kb upstream to 1 kb upstream). To complement these sets of sequences, we also retrieved proximal promoters and enhancers elsewhere in the genome that are not bound by MyoD. Thus, we have collected four sets of DNA sequences for this analysis. Analysing them, we observed some remarkable differences between the proximal and distal binding regions in terms of preferred factors.

Binding sites of many factors not involved in myogenesis or previously established to be associated with MyoD (like CDP, Evi, Oct, RORalpha, SRY, E2F) are not detected in a considerable numbers in the close proximity of MyoD in the ChIP-Seq bound sequences (27). Instead, these factors’ binding sites are enriched in sequences not bound by MyoD. This may signify that the binding of these factors in close proximity to MyoD is restricted by the innate properties of the MyoD-bound sequences. Another, non-mutually exclusive possible interpretation is that these factors in MyoD-unbound sequences either block or restrict the binding of MyoD around their binding sites.

In our analysis, we found factors like Meis1, E47, AP-4 to be associated to the predicted MyoD-binding sites in both bound and unbound sequences. In the context of myogenesis, this combination is functional; therefore, we can expect the association in the MyoD bound sequences. However, the occurrence of the association of these factors with MyoD in the unbound sequences is unexpected. This might occur because of the false discovery of the binding sites with computational methods.

From this investigation, it is clear that the binding of MyoD to its binding sites is associated with the presence or absence of binding sites for other factors. The relationship of these factors’ binding sites can be taken into account to distinguish or discriminate the functional MyoD-binding sites from the non-functional ones. For example, considering the limited binding sites of the aforementioned factors like Oct, CDP and so forth around MyoD-binding sites can enhance the discrimination of the functional MyoD-binding sites from that of non-functional ones. Further, we have analyzed the association of MyoD with other factors with respect to the TSS, i.e. in proximal and distal bound sequences.

As we have both the bound and not bound sequences, we could calculate the enrichment score for each of the factors if they are differentially bound in either of these regions. The enrichment score for each factor is calculated by dividing the number of promoters having the factor co-occurring with MyoD in the bound sequences by the number of promoters having the same factor co-occurring with MyoD in unbound sequences. Likewise, from these bound and not bound sequences, we measured the significance in terms of z-score. We regard the number of promoters having a factor co-occurring with MyoD in bound sequences as the observed occurrence and the number of promoters having the same factor co-occurring with other E-box binding factors in unbound sequences as background occurrence.

Thus, we obtained enrichment score and z-score for each factor. Based on these scores, we rank each of the factors in ascending order. Thus, we have two lists of factors, sorted in ascending order with respect to the scores. This analysis is done for both the proximal sequences and for distal sequences separately.

We selected the top ranked factors from both the proximal and distal sequences; those that have congruent ranks with both scoring methods (rank differences of 10 or less) are reported in Tables 7 and 8. From this analysis, we have seen a difference in the preference of factors around MyoD-binding sites in proximal and distal bound regions. In proximal bound sequences, the factors enriched are AP-2, CREB, USF, ETF and E2F (Table 7). However, in the enhancer, the top ranking factors are found to be E-box proteins, such as AP-4, LBP-1 and HEN1 (Table 8). The other factors enriched in the bound enhancers are NRSF, SZF1-1, SP1 and so forth (Table 8). The observations from this analysis have the potential to help make better predictions of functional MyoD binding sites.

**Table 7.** The factors with higher ranking in proximal sequences

ID	Name	Pattern	Rank	
			Significant	Enrichment
M00469	AP-2alpha	GCCNNNRGS	1	2
M00470	AP-2gamma	GCCYNNGGS	2	4
M00740	Rb:E2F-1:DP-1	TTTSGCGC	3	1
M00938	E2F-1	TTGGCGCGRAANNGNM	4	3
M00177	CREB	NSTGACGTAANN	5	5
M00189	AP-2	MKCCCSCNGGCG	6	8
M00121	USF	NNRYCACGTGRYNN	7	6
M00516	E2F	TTTSGCGCGMNR	8	7
M00113	CREB	NNGNTGACGTNN	9	9
M00695	ETF	GVGGMGG	10	13

The table shows the top ranked factors from the proximal promoters and those that have congruent ranks in both scoring schemes (enrichment score and the z-score of co-occurrence of TFBS with MyoD). The factors in the table are sorted in ascending order of the scores. The factors are selected based on top rank (above 100) and the differences between the ranks of the scoring schemes by 10 or less.

**Table 8.** The factors with higher ranking in distal sequences

ID	Name	Pattern	Rank	
			Significant	Enrichment
M00927	AP-4	RNCAGCTGC	1	5
M01287	Neuro	CAGCTG	2	12
M00644	LBP-1	CAGCTGS	3	1
M00256	NRSF	TTCAGCACCACGGACAGMGCC	11	8
M01109	SZF1-1	CCAGGGTAWCAGCNG	12	4
M00068	HEN1	NNNGGNCNCAGCTGCGNCCCNN	23	23
M01256	REST	NNNNGGNGCTGTCCATGGTGCT	30	34
M00173	AP-1	RSTGACTNANW	33	42
M00979	Pax-6	CTGACCTGGAACTM	42	45
M01175	CKROX	SCCCTCCCC	45	47
M00480	LUN-1	TCCCAGCTACTTTGGGA	46	52
M00257	RREB-1	CCCCAAACMMCCCC	47	49
M00933	Sp1	CCCCGCCCCN	48	55
M00072	CP2	GCHCDAMCCAG	53	43
M00378	Pax-4	NNNNNYCACCCB	63	67
M00721	CACCC-binding	CANCCNNWGGGTGDGG	65	58
M01105	ZBRK1	GGGSMGCAGNNNTTT	72	73
M00687	alpha-CP1	CAGCCAATGAG	79	74
M00765	COUP	TGACCTTTGACCC	83	80
M00794	TTF-1	NNNNCAAGNRNN	91	86

The table shows the top ranked factors from the enhancers and those that have congruent ranks in both scoring schemes (enrichment score and the z-score of co-occurrence of TFBS with MyoD). The factors in the table are sorted in ascending order of the scores. The factors are selected based on top rank (above 100) and the differences between the ranks of the scoring schemes by 10 or less.

## DISCUSSION

As mentioned formerly, the mapping of the location of TFBSs is error prone, but the inclusion or modeling of additional type of information has the potential to mitigate this weakness. Additional information can be the accompanying factors with the MyoD-binding sites. For instance, the affinity or aversion of MyoD to bind to certain sequences depends on the surrounding factors. As indicated previously by (27), the co-occurrence of E-box proteins can be used to improve the identification of functional MyoD-binding sites from the genomic sequences. Likewise, in this study, we have identified factors that can also be included into the model that will further strengthen the efficiency to discriminate the functional binding sites from that of the nonfunctional ones.

We found out the distribution of the factors with respect to their mutual occurrence and the distance between them. From this *in silico* information, we calculated the typical pair-wise distance between each two TFs and extracted the combinations of TFs within certain interval with respect to one particular TF along the promoters of the human genome. This analysis is further extended to determine whether there is any preferential positioning between the selected combinations.

In this present work, we obtained the relationship of binding sites of different factors with respect to MyoD in terms of the co-occurrence and mutual positioning. The distribution of the aforementioned factor’s binding sites around MyoD is non-trivial and as indicated in previous studies, some of them are well established for their cooperation with MyoD during myogenesis. Most factors having important role in myogenesis and recruited together with MyoD are found to be present within the range of ±100 bp.

Recently Guo *et al.* (72) have developed a method named GEM to determine the pair-wise spatial binding constrain of TFs *in vivo*. In their study, they have also focused on the aspect of the appropriate spacing between the TFBS. However, their method is different from our approach. In Guo *et al.*’s approach, they first computationally discover the motifs for TFs whose binding motifs are not available in public databases. In our approach, we have taken the TF-binding motifs derived experimentally and determined the functional binding sites on the sequences from the ChIP experiment for specific TF to determine the mutual positioning among the associated factors.

In addition to these, we found some other TFBSs co-occurring distinctly in a vicinity of MyoD. The presence of the other non-myogenic or not muscle-specific factors around MyoD may explain the involvement of regulation of a large number of genes by MyoD. Positional preference of MyoD with certain factors with different tissue distributions may be related to the regulation of many genes by MyoD in a different tissue-specific manner. For example, in our analysis, we detected Egr having a preferential position with the MyoD in both the data sets (human promoters from DBTSS and ChIP-Seq MyoD bound sequences). The expression of certain members of the Egr gene family is also detected in the C2C12 cells (71), like Egr1-3. This suggests a role of Egr proteins in muscle development*.* Furthermore, from the expression profiling data, we could see that Egr1, Egr2 and Egr3 gene expression is upregulated during myoblast differentiation, suggesting a possible role of these proteins in cooperating with MyoD. In the partitioned ChIP-Seq data (27), the binding motif for this factor is found to be enriched in the proximal promoters.

Similarly, the factors playing important roles in non-muscle tissues like orphan steroid receptor ARP-1 and Ikaros TF (found to play critical functions in the control of lymphohematopoesis and immune regulation) are found to be mutually positioned with MyoD and also found to be expressed in C2C12 cells (71). These interactions may suggest that some TFs not related to muscle development also cooperate with MyoD or, more likely, with factors with MyoD-like binding patterns.

In our comparative co-occurring TF in myoblast and myotubes, we observed that certain TF prefers to bind at close proximity to MyoD in myoblast but also at distance in myotubes. For example, SMAD3 in Table 6, in myoblast, the mutual position is at ±5 bp, but in myotubes, the preferred mutual position is stretched up to 60 bp upstream and 86 bp downstream along with the close mutual preferred positions. We have no explanation to this phenomenon. This indicates that there are many targets of MyoD that are myoblast or myotubes specific. It could be that the circumstances are such in the condition-specific targets that the optimal binding distance for the partners of MyoD is different.

Apart from this, there is another interesting observation that can be found from Table 6. For instance, the BSs for NERF1a in myoblast are mostly in downstream of the MyoD BS, whereas the BSs of NERF1a in myotubes are mostly in upstream. However, the distance from MyoD is almost conserved in myoblast and myotubes. Only the direction of the preferred position is reversed. This effect can also be seen with NF-E2. The preferred BSs NF-E2 in myoblast is mostly in upstream, whereas in myotubes, the preferred BSs are mostly distributed in downstream.

As observed from our analysis, the binding sites of the various factors are distributed symmetrically around MyoD. As we included both the strands in our study, the question may be raised whether the DNA strand has any effect on the distribution of the TFBSs with respect to the MyoD-binding sites. To examine this, we mapped the TFs from the Table 1–5 in the single strand of the promoters. Even in this analysis, we could see the positional preference of BSs with respect to the MyoD BS in addition to the overlapping preferences. However, the peak of the distribution is reduced in the single strand analysis, which is expected. But some of the factors, which have positional preferences in positions both upstream and downstream of MyoD in both strand analysis, are found to have preferences only in upstream or downstream direction when they are mapped in a single strand. This means that one of the preferred positions is contributed by the other strand. This may also imply that these binding sites have strand specificity in addition to the positional preferences.

In our analysis, we found the occurrence of NeuroD (primarily regulates neuronal differentiation) in the overlapping position with MyoD like that of the E-protein. MyoD dimerizes with E proteins; however, MyoD does not dimerize with NeuroD, and thus the occurrence of NeuroD in the overlapping position with MyoD is merely because of the motif pattern CANNTG. The PWM for NeuroD in our analysis from TRANSFAC has the consensus motif CAGCTG, which is similar to the MyoD motif from TRANSFAC, CACCTG.

As mentioned earlier, the detection of functional binding sites is error prone, which can lead to MyoD being mistakenly identified as E-box proteins or vice versa. However, incorporating or considering the additional information like the presence and absence of certain factors in close proximity of the factor of interest of study can increase the efficiency of the binding site identification algorithm. For example, if the MyoD-binding site is detected close to factors like E-box proteins or AML1 and not in close proximity to the factors like CDP or GR, the detected binding site can be regarded as functional if such criteria are implemented.

In this study, we could find many of the factors associated with MyoD; however, this kind of study is still limited by the paucity of binding site information for many TFs. The site-specific information for many factors is not yet available in the library of TFs like TRANSFAC.

For instance, from previous studies, it is known that Pax7-mediated activation of MyoD specifies the population of muscle stem cells that enter the differentiation program (73,74). In our analysis, the occurrence of Pax6 and Pax4 instead of Pax7/Pax3 motif could be false-positive discovery because of their similar motif pattern as defined in the TRANSFAC database. Therefore, we cannot deny the discovery of the false-binding sites when we consider the TFBSs database, which is not mature enough. Nevertheless, based on the existing site-specific information and the statistical analysis of over-representation of some factors with MyoD, we could establish the association of some factors, which were not previously studied, and these factors can be used to discriminate between the functional and the non-functional MyoD-binding sites. This study also emphasizes the preference of a factor binding sites with respect to the TSS, which can be used to build a model to help in identifying real binding sites in the genome.

## SUPPLEMENTARY DATA

Supplementary Data are available at NAR Online.

## FUNDING

The Canada Fund for Innovation Leaders Opportunity Fund/Ontario Research Foundation [Grant 22880 to I.I.]; and National Science and Engineering Research Council [grant GPIN/372240-2009 to I.I. and S.N.]. Funding for open access charge: University of Ottawa.

*Conflict of interest statement.* None declared.

## Supplementary Material

Supplementary Data
