# Supplementary material for: Identification of cis-regulatory modules in promoters of human genes exploiting mutual positioning of transcription factors
Source: Nucleic Acids Res. 2013 Aug 2;41(19):8822–41. doi: 10.1093/nar/gkt578 (PMC3799424; doi:10.1093/nar/gkt578)
Supplement: Supplementary Data [file supp_41_19_8822__index.html]

Identification of cis-regulatory modules in promoters of human genes exploiting mutual positioning of transcription factors — Identification of cis-regulatory modules in promoters of human genes exploiting mutual positioning of transcription factors — Supplementary Data 

# Identification of cis-regulatory modules in promoters of human genes exploiting mutual positioning of transcription factors

## 

files

**Files in this Data Supplement:**

- Supplementary Data - pdf file
