# Supplementary material for: Identification of cis-regulatory modules in promoters of human genes exploiting mutual positioning of transcription factors
Source: Nucleic Acids Res. 2013 Aug 2;41(19):8822–41. doi: 10.1093/nar/gkt578 (PMC3799424; doi:10.1093/nar/gkt578)
Supplement: Supplementary Data [file supp_gkt578_nar-03421-n-2012-File005.pdf]

Supplementary Figure 1:**The figure shows the distribution of all the obtained z-score.**

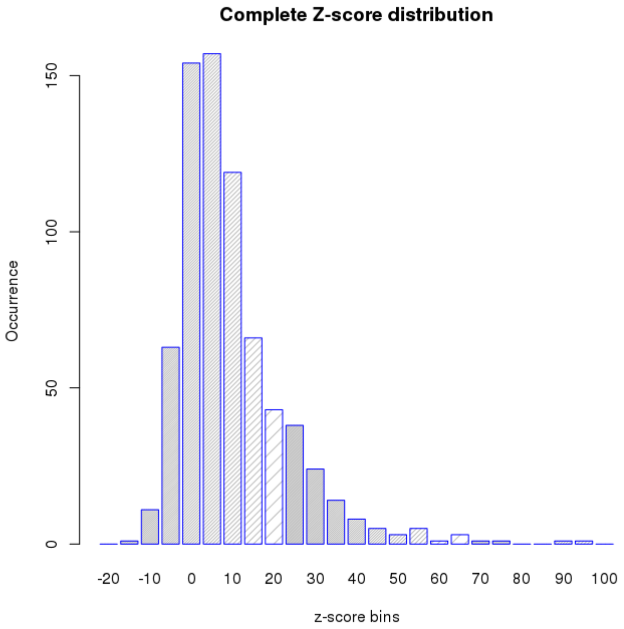

Supplementary Table 1: **These factors are found to be significantly co-occurring with MyoD within ±100bp because of their similar or identical motif pattern.**

TF name	TRANSFAC id	No of promoters with z-score	Consensus from TRANSFAC	Significant positions with p-values
E2A	M00973	5221(52.74)	CACCTGNY	-1(<2.2e-16)<br>1(<2.2e-16)
Myogenin	M00712	3599(55.08)	RGCAGSTG	-1(<2.2e-16)<br>1(<2.2e-16)
E12	M00693	3468(33.01)	RRCAGGTGNCV	-1(<2.2e-16)<br>0(<2.2e-16)
TAL1	M00993	2654(40.02)	TCCAKCTGNY	0(<2.2e-16)
Ebox	M01034	2463(47.26)	NCACSTGNCN	-1(<2.2e-16)<br>1(<2.2e-16)
Lmo2	M00277	1804(25.65)	CNNCAGGTGBNN	0(<2.2e-16)<br>49(=9.046e-12)
E47	M00071	1220(16.31)	NNNMRCAGGTGTTMNN	0(<2.2e-16)<br>16(=1.936e-10)<br>33(<2.2e-16)
NeuroD	M01288	972(26.69)	NNSCWGCTGNSY	-3(<2.2e-16) 0(<2.2e-16)

				3(<2.2e-16)
LBP-1	M00644	964(28.11)	CAGCTGS	-1(<2.2e-16) 0(<2.2e-16)
Tal-1alpha:E47	M00066	871(7.77)	NNNAACAGATGKTNNN	0(<2.2e-16)
AP-4	M00927	840(9.85)	RNCAGCTGC	-1(<2.2e-16) 0(<2.2e-16)
HEB	M00698	814(18.59)	RCCWGCTG	-2(<2.2e-16)1(<2.2e-16)<br>2(<2.2e-16)
Tal-1beta:E47	M00065	724(5.52)	NNNAACAGATGKTNNN	0(<2.2e-16)
Tal-1beta:ITF-2	M00070	703(6.77)	NNNAACAGATGKTNNN	0(<2.2e-16)
AREB6	M00413	642(18.08)	WNWCACCTGWNN	-33(<2.2e-16)<br>0(<2.2e-16)<br>16(=4.968e-12)<br>33(<2.2e-16)

Supplementary Table 2: **Summary of overlap between MyoD binding sites and those of other TFs, as discovered by ChIP-seq**, reported in (27,55-57).

**Cells**	**Method**	**TF**	**Type**	**Number of BS**		**Number and % of MyoD BS that overlap with BS of X**	**Ref.**
				**X**	**MyoD**		
Primary	ChIP-seq	Tag-Snail		5,465	9,300	442 – 4,8%	(27)
C2C12	ChIP-seq	c-Jun	AP-1	18,712	23,271	5,961 – 25,6%	(55,56)
C2C12	ChIP-seq	Msx1	Homeo	62,225	23,271	9,837 – 42,3%	(55,57)
C2C12	ChIP-seq	E2F4	E2F	4,546	13,040	248 – 1.9 %	Caltech, mouse ENCODE
C2C12	ChIP-seq	SRF	AT-rich	2,610	13,040	485 – 3.7%	Caltech, mouse ENCODE
C2C12	ChIP-seq	CTCF	CCCTC	21,117	13,040	539 – 4.1%	Caltech, mouse ENCODE

C2C12	ChIP-seq	NRSF	NRSF	15,127	13,040	662 – 5.1%	Caltech, mouse ENCODE
C2C12	ChIP-seq	USF1	E-box	8,579	13,040	1,475 – 11.3%	Caltech, mouse ENCODE
C2C12	ChIP-seq	CEBPbeta	CAAT	11,434	13,040	2,213 – 17%	Caltech, mouse ENCODE
C2C12	ChIP-seq	Max	E-box	8,930	13,040	2,488 – 19.1%	Caltech, mouse ENCODE
C2C12	ChIP-seq	FosL1	AP1	6,507	13,040	2,614 – 20.0%	Caltech, mouse ENCODE
C2C12	ChIP-seq	Myog	E-box	24,360	13,040	4,394 – 33.7%	Caltech, mouse ENCODE
C2C12	ChIP-seq	TCF12	E-box	16,467	13,040	6,973 – 53.5%	Caltech, mouse ENCODE

Supplementary Table 3: **The table summarizes the proportion of the promoters having factors co-occurring with MyoD in classified promoters based on CpG islands.**

Name	Transfac ID	Proportion of promoters having MyoD+factor		
		CpG+	CpG-	Combined
Kid3	M01160	0.183862	0.168371	0.172845
E2A	M00973	0.163458	0.152197	0.162922
myogenin	M00712	0.125648	0.114073	0.112307
E12	M00693	0.123112	0.0970006	0.108219
TAL1	M00993	0.0809222	0.0853194	0.0828184
Ebox	M01034	0.074928	0.0722691	0.0768583
Lmo2	M00277	0.0742363	0.0519875	0.0562941
ELF1	M01266	0.0517579	0.0518164	0.0504899
ZNF333	M01230	0.0340058	0.0481794	0.0439056
E47	M00071	0.0419597	0.0413333	0.0380703
NFAT1	M01281	0.0327378	0.0362843	0.0350434
Ikaros	M01169	0.0239769	0.0348295	0.0323597
AREB6	M00414	0.0300865	0.0307646	0.0314236
NeuroD	M01288	0.0405764	0.0340593	0.0303314
LBP-1	M00644	0.0463401	0.0368833	0.0300818
Lyf-1	M00141	0.0189049	0.0308502	0.0282407

AML1	M00751	0.0182133	0.031492	0.0281782
AML1a	M00271	0.0182133	0.031492	0.0281782
HOXA13	M01292	0.0189049	0.0318771	0.0279598
Tal-1alpha:E47	M00066	0.0278963	0.0295238	0.0271797
MAFB	M01227	0.0278963	0.0261435	0.0263059
AP-4	M00927	0.0455331	0.0388944	0.0262123
PPARG	M01270	0.0160231	0.0286252	0.0255882
HEB	M00698	0.0319308	0.0294382	0.025401
HNF4	M01032	0.0185591	0.0267853	0.0246833
T3R	M00963	0.0199424	0.0304651	0.0246833
TEF-1	M00704	0.0236311	0.0249027	0.0235911
Pitx2	M00482	0.0149856	0.0249454	0.0229358
Tal-1beta:E47	M00065	0.0223631	0.0237046	0.0225925
LEF1	M00805	0.0202882	0.0235334	0.022062
Tal-1beta:ITF-2	M00070	0.0216715	0.0255017	0.0219372
ARP-1	M00155	0.0130259	0.0227633	0.0206266
PKNOX2	M01411	0.0200576	0.0224209	0.0204394
AREB6	M00413	0.0146398	0.0212657	0.0200337
AP-4	M00005	0.020634	0.0206666	0.0195344
MAZ	M00649	0.0238617	0.0172864	0.0191288
HOXA4	M00640	0.00945245	0.0218647	0.0190975
ETS2	M01207	0.0187896	0.0201532	0.0190663
PREP1	M01459	0.0137176	0.0186128	0.0169132
TBX5	M01044	0.010951	0.0189979	0.016882
CKROX	M01175	0.0262824	0.0130076	0.0166635
PU.1	M00658	0.0165994	0.0169869	0.0164139
Meis2	M01488	0.0154467	0.0175859	0.0162579
MEIS1	M00419	0.0153314	0.0161311	0.0162579
SREBP-1	M00220	0.0102594	0.0175431	0.0160706
Sp1	M00933	0.0272046	0.0115956	0.0159146
Pax-4	M00377	0.00783862	0.0181421	0.0158522
MEF-2	M00233	0.00876081	0.0174575	0.015665

Supplementary Table 4: **The table summarizes the factors significantly co-occurring with MyoD (z-score > 3).But no positions were detected to be significant with the z-score above 10.**

**TF name**	**TRANSFAC id**	**No of promoters**	**Consensus from TRANSFAC**
Meis1	M01419	498	WANNASCTGTCAAWNN
STAT5A	M00499	497	NNNTTCYN
SREBP2	M01177	467	NNGYCACNNSMN
TFII-I	M00706	461	RGAGGKAGG
Ets	M00971	459	ACTTCCTS
MRG2	M01395	440	NANNASCTGTCAANNN
ETF	M00695	438	GVGGMGG
Sp1	M00931	424	GGGGCGGGGC
COUPTF	M01036	422	NNNNNTGACCYTTGNMCNYNGMN

TGIF2	M01407	420	AANNAGCTGTCAAWNN
PU.1	M01172	418	NNNNYYYACTTCCTCTTTY
NERF1a	M00531	414	YRNCAGGAAGYRNSTBDS
SP4	M01273	405	SCCCCGCCCCS
ATF6	M00483	400	TGACGTGG
Nanog	M01247	393	NNWNNANAACAAWRGNNNNN
AP-2rep	M00468	391	CAGTGGG
RREB-1	M00257	388	CCCCAAACMMCCCC
LF-A1	M00646	388	GGGSTCWR
BLIMP1	M01066	385	AGRAAGKGAAAGKR
KROX	M00982	377	CCCGCCCCCRCCCC
CTCF	M01259	373	NNNNNNCCASNAGRKGGCRS
E2F6	M01252	363	CNTTTCNT
STAT6	M00494	349	NAWTTCCN
NURR1	M01269	344	YRRCCTT
TTF-1	M00794	341	NNNNCAAGNRNN
SMAD4	M00733	337	GKSRKKCAGMCANCY
IRF	M00972	336	RAAANTGAAAN
ESE1	M01214	336	DRYTTCCTGW
SREBP	M01168	333	NNNNYCACNCCANNN
NUR77	M01217	326	NTGACCTTBN
ATF5	M01295	319	CYTCTYCCTTA
Elf-1	M00746	316	RNWMBAGGAART
RP58	M00532	312	NNAACATCTGGA
HNF4,	M00967	309	AARGTCCAN
PPAR,	M00762	308	RGGNCAAAGGTCA
CACCC-binding	M00721	307	CANCCNNWGGGTGDGG
Sp3	M00665	305	ASMCTTGGGSRGGG
STAT	M00777	303	NNNNNTTCTKGGA
USF2	M00726	300	CASGYG
Tel-2	M00678	298	YTACTTCCTG
SMAD3	M00701	295	TGTCTGTCT
NRSF	M00256	295	TTCAGCACCACGGACAGMGCC
HIC1	M01072	294	NSNNNNTGCCCSSNN
ZNF219	M01122	293	CRCCCCCCNCCC
ISGF-3	M00258	285	CAGTTTCWCTTTYCC
PPARalpha:RXRalpha	M00518	282	NNRGGTCATWGGGGTSANG
PR,	M00960	281	NWNAGRACAN
SP1:SP3	M01219	280	CCSCCCCCYCC
GR	M00205	280	GGTACAANNTGTYCTK
BCL6	M01183	272	YNNNNNRNWNNNYTTT
AP-2	M00915	271	SNNNCCNCAGGCN
STAT1	M01260	270	NNTTTCYNGGAARNNNNNNNN
HMGIY	M01010	269	NNKKNAWTTTNYTNN
HNF3	M01012	264	NNNNNTRTTTRYTYWNKN
CP2/LBP-1c/LSF	M00947	264	GCTGGNTNGNNCYNG
deltaEF1	M00073	261	NNNCACCTNAN
ZBRK1	M01105	260	GGGSMGCAGNNNTTT
p300	M00033	260	NNNGGGAGTNNNNS

FLI1	M01208	260	CMGGAWGTSMN
Egr	M00807	259	GTGGGSGCRRS
FOXJ2	M00422	253	NNNWAAAYAAAYANNNNN
FAC1	M00456	250	NNNCAMAACACRNA
LRH1	M01142	248	NNRRCCTTGVAN
IRF3	M01279	247	NBNBTTTCSCTTT
P50:RELA-P65	M01224	246	GGANTTYCCCWN
Pax	M00808	244	CTGGAACTMAC
EBF	M00977	244	KTCCCYWGRGA
POU5F1	M01307	243	NATGCAANNN
NF-E2	M00037	241	TGCTGAGTCAY
NF-kappaB	M00774	240	NNNNKGGRAANTCCCN
FOXO3A	M01137	240	TRTAAACAANWN
ZID	M00085	239	NGGCTCYATCAYC
NF-muE1	M00651	239	CGGCCATCT
FOXD3	M00130	239	NAWTGTTTRTTT
WT1	M01118	238	SMCNCCNSC
KAISO	M01119	236	NTCCTGCNAN
GATA-4	M00632	236	AGATADMAGGGA
CP2	M00072	236	GCHCDAMCCAG
ERR	M00511	235	NNNTNAAGGTCANN
NF-1	M00806	234	NTGGNNNNNNGCCAANN
FOX	M00809	232	KWTTGTTTRTTTW
TEF	M01305	231	YACATTCCWSNG
Nrf-2	M00821	230	NTGCTGAGTCAKN
TBX5	M01019	226	NNAGGTGTNANN
HNF3alpha	M00724	226	TRTTTGYTYWN
Olf-1	M00261	225	NNCDABTCCCYAGRGARBNKGN
LRF	M01100	225	VNNRMCCCC
COUP	M00765	225	TGACCTTTGACCC
Hand1:E47	M00222	220	NNNNGNRTCTGGMWTT
Zic2	M00449	218	KGGGTGGTC
GCNF	M00526	217	NTCAAGKTCAAGKTCANN
HFH3	M00289	214	KNNTRTTTRTTTA
RelB:p52	M01239	213	GGGGNTTTCC
P50:P50	M01223	210	NRGGGAMTNCCCN
c-Myc	M01145	210	RACCACGTGCTC
AP-2gamma	M00470	206	GCCYNNGGS
ER	M00959	205	NAGGTCANNNY
NF-AT	M00935	204	NWGGAAANWN
Freac-7	M00293	203	WNNANATAAAYANNNN
SPI-B	M01204	201	AAAAWGMGGAAGTWNSN
FRA1	M01267	199	TGAGTCAN
C/EBPgamma	M00622	199	CTBATTTCARAAW
Six-6	M01398	198	NATRGGGTATCANNWNT
Pax-2	M00486	198	NNNAAASNN
c-Rel	M00053	197	SGGRNTTTCC
HSF	M00641	196	TTCCMGARGYTTC
Staf	M00262	195	NTTWCCCANMATGCAYYRCGNY
ICSBP	M00699	195	RAARTGAAACTG
STAT1:STAT1	M01212	194	NTTTCCMGGAAAA

Six-1	M01313	194	NNNRGGGTATCANNNNT
HNF3beta	M00131	194	KGNANTRTTTRYTTW
Gfi-1	M00250	194	NNNNNNNAAATCACWGYNNNNNNN
BRCA1:USF2	M01082	194	KTNNGTTG
alpha-CP1	M00687	194	CAGCCAATGAG
Helios	M01004	193	WNWAGGAAAAN
GLI	M01037	193	NBTGGGTGGTCN
IRF-7	M00453	192	TNSGAAWNCGAAANTNNN
GABP	M01258	192	CACTTCCGGNNN
DBP	M00624	191	AGCAHAC
SOX4	M01308	190	AACAAWGR
PEA3	M00655	190	ACWTCCK
PPARgamma:RXRalpha	M00512	189	NNWGRGGTCAAAGGTCANNNN
LEF1	M01022	189	NWTCAAAGNN
HFH4	M00742	189	AWKTGTTTGTTTA
AML	M00769	189	NNGKNTGTGGTTWNC
Pax-5	M00143	187	BCNNNRNGCANBGNTGNRTAGCSGCHNB
FOXO4	M00472	187	RWAAACAANNN
PEBP	M00984	184	GNTNACCACARANNK
Oct-1	M00342	184	TATGCAAATN
GATA-3	M00077	184	NNGATARNG
FOXO1	M00473	184	NRWAAACAAN
IRF-2	M00063	181	GAAAAGYGAAASY
IRF-1	M00062	181	SAAAAGYGAAACC
Octamer	M00795	180	TNATTTGCATN
TATA	M00216	178	NCTATAAAAR
SOX10	M01131	178	CWTTGTN
MYB	M00913	178	NAACNGNCN
MAZR	M00491	178	NSGGGGGGGGMCN
TFIIA	M00707	177	TMTRWRAGGRSS
STAT3:STAT3	M01220	177	TTKMCGGGAAMTSC
FOXM1	M00630	177	ARATKGAST
TCF11:MafG	M00284	175	NNNNNATGACTCAGCANTTNNG
PPARalpha:RXRalpha	M00242	175	CARAACTAGGNCAAAGGTCA
TCF-4	M00671	174	SCTTTGAW
SRF	M01257	174	NNCCAWAWAAGGNMANNN
SOX2	M01272	174	NNNNCCWTTGTTWTKN
Freac-3	M00291	174	NNNNNGTAAATAAACA
HMG	M00750	173	GGAAAWT
AP-2alphaA	M01045	171	ANNGCCTNAGGSNNT
Osf2	M00731	170	ACCACANM
TBX18	M01262	169	AGGTGTGAAATTCGCACCT
STAT4	M00498	168	SNWTTCNN
Cdx	M00991	167	NAYNRHNARAKTHATAAA
RUSH-1alpha	M01107	165	NNMCWTNKNN
HFH8	M00294	165	NNNTGTTTATNTR
RFX	M00975	164	SHGTTGCSN
TFE	M01029	163	TCATGTGN
PARP	M01211	163	YDRGAAAWAS
OTX	M01117	163	GATTANNT

SRY	M00160	161	NWWAACAAWANN
OCT-x	M00210	161	CTNATTTGCATAY
c-Ets-1	M01078	161	NNNRCCGGAWRYNNNN
aMEF-2	M00403	161	CKGDYTAAAAATAACYMM
MEF2A	M01301	160	RGYTATTTTTAR
Myc	M00799	159	CACGTGS
MIF-1	M00279	159	NNGTTGCWWGGYAACNGS
NF-Y	M00209	157	NCTGATTGGYTASY
p53	M00761	156	RGRCAWGNCY
HNF4alpha	M00638	156	VTGAACTTTGMMB
TRF1	M01237	155	YTAGGGTTAGGGTTR
Nkx2-1	M01312	155	NNANCCACTTGAMNTT
HNF3A	M01261	155	RWGTAAACAN
C/EBPbeta	M00109	155	RNRTKDNGMAAKNN
ATF3	M00513	154	CBCTGACGTCANCS
NKX25	M01414	153	NNANCCACTTRAAWTT
AR	M00953	152	NNNGNRRGNACANNGTGTTCTNNNNNN
Zec	M01081	151	CAAGGTTGGTTGC
VDR	M00961	151	CNSNNTGAACCN
MEIS1B:HOXA 9	M00421	150	TGACASTTTWAYRR
FOXP3	M00992	149	RWARTGTTGTTTCAKAC
RXR	M01152	148	NNAGTTCA
Elk-1	M00025	148	NNNNCCGGAARTNN
AP-2alpha	M00469	148	GCCNNNRGS
Nkx6-1	M00424	147	TWTTTAATTGGTT
HSF2	M01244	147	GAANRTTCTAGAA
C/EBP	M00770	147	NNNTKNNGNAAN
Zic1	M00448	146	KGGGTGGTC
mTERF	M01245	146	TGGTARWRGTYGGT
six4	M01374	145	ANNNATGACACCNNNNN
MTF1	M01242	145	GTGTGCANMACTTTGCGCAC
SOX	M01014	144	CTCTTTGTTANGA
GAF	M01209	144	CRNATTCCYNK
AP-1	M00199	144	NTGASTCAG
MEIS1A:HOXA 9	M00420	143	TGACAGKTTTAYGA
RBP-Jkappa	M01112	142	ANCGTGGGAAM
NGFI-C	M00244	142	WTGCGTGGGYGG
GATA-1	M00128	142	NNCWGATARNNNN
ATF1	M00691	141	CYYTGACGTCA
ELF5	M01197	140	WNAAGGAARTW
Barhl2	M01446	139	NAMAANCAATTAANNN
RSRFC4	M00026	138	RNKCTATTTWTAGMWN
CHOP:C/EBPa lpha	M00249	138	NNRTGCAATMCCC
Tst-1	M00133	137	NNKGAATTAVAVTDN
c-Myb	M00183	137	NNNAACTGNC
C/EBPdelta	M00621	136	MATTKCNTMAYY
USF	M00122	135	NNRNCACGTGNYNN
Pit-1	M00802	135	NMTTCATAAWTATWNMNA
GATA	M00789	135	WGATARN

GATA-6	M00462	135	NNNGATWANN
FOXO3	M00477	134	TNNTTGTTTACNTW
Gfi1b	M01058	133	TAAATCACTGCW
Roaz	M00467	132	GCACCCAWGGGTGM
Oct-2	M01368	131	NNNTATGCAAATNNNN
Nkx3-2	M01482	131	NNNAACCACTTAANNAN
HOXA7	M01108	131	YCAATCT
c-Ets-1(p54)	M00074	131	NNACMGGAWRTNN
Bach2	M00490	131	SRTGAGTCANC
HES1	M01009	130	ANSBCKYGTGSCNNG
HOXA10	M01464	129	NNNGYAATAAAATNNW
Pax-8	M00328	128	NCNNTNNTGCRTGANNNN
PLZF	M01075	126	KTNNWTNGNNGNTAAAGYTTKATYWGTTC
STAT5B	M00459	125	NAWTTCYNGGAAWTN
RFX1	M00281	125	NNGTNRCNATRGYAACNN
IRX4	M01410	124	WNNNWACATGTWAWANN
HOXD13	M01404	124	NNNYYAATAAAANNNN
Bach1	M00495	124	NNSATGAGTCATGNT
VDR:RXR	M01202	123	GGGTCAWNGRGTTCA
RORalpha	M01138	123	WAANTRGGTCA
STATx	M00223	122	TTCCCGKAA
GCM	M00634	122	CNNRCCCGCATD
E2F1	M01250	122	CGTTTCNN
AhR	M00139	122	CCYCNRRSTNGCGTGASA
hoxa9	M01351	121	NCGGYCATWAAAWTANW
Egr-2	M00246	121	NTGCGTRGGCGK
GATA-2	M00348	120	NNWGATAASA
FXR	M01268	119	AGGTCAN
Irx2	M01405	118	WNNNTACATGTANNWNW
RORalpha2	M00157	117	NWAWNTAGGTCAN
ATF4	M00514	117	CVTGACGYMABG
HOXB9	M01426	116	NRRGCMATAAAANNNN
FXR/RXR-alpha	M00631	116	CARGKTSAWTRACC
TBX22	M01195	115	AGGTGTGAAWTTSTCACCT
HNF6	M00639	115	HWAAATCAATAW
SOX5	M00042	114	NNAACAATNN
RORalpha1	M00156	114	NWAWNNAGGTCAN
POU2F3	M01476	114	NNNTATGCAAATNNNN
Nkx5-1	M01413	114	NNNAGCAATTAANNNAT
GATA-X	M00203	114	NGATAAGNMNN
Freac-2	M00290	114	NNANNGTAAACAANNN
Alx-4	M00619	113	CCTGAGAATAATC
Nkx6-2	M00489	112	NWADTAAWTANN
C/EBPalpha	M00116	112	NNATTRCNNAANNN
DMRT1	M01146	111	TTGMTACAWTGTTKC
TCF11	M00285	109	GTCATNNNWNNNNN
Otx1	M01366	108	NNNRGGGATTAATWNNN
Irx5	M01472	108	NNNNWACATGTWNNWNW
c-Myc:Max	M00118	108	NNACCACGTGGTNN
POU6F1	M01479	107	NNNNWTAATGAGSTNNN

AFP1	M00616	106	ATTAAYTRCAC
ZABC1	M01306	105	ATTCCNAC
Freac-4	M00292	105	CTWAWGTAAACANWGN
Barhl-1	M01332	105	NANAACCAATTARNNN
SOX9	M00410	104	NNNNAACAATRGNN
AP-3	M00690	104	TCYMMATT
POU1F1	M00744	103	ATGAATAAWT
Ncx	M01420	102	NAATNAATTAATAANWW
Lmx-1	M01409	102	CNNNTTAATTAAWWNNN
Brn-2	M00145	102	NNCATNSRWAATNMRN
Arnt	M00539	102	NNNNNRTCACGTGAYNNNNN
Otx3	M01403	101	NNRWNMGGATTANKNNN
Cdx-1	M01373	101	NNNGGTMATAAAANNN
POU6F1	M01462	100	NNNNATAATGAGSTNNN
Egr-3	M00245	99	NTGCGTGGGCGK
POU3F2	M00463	98	ATGMATWWATTCAT
Nkx2-2	M00485	98	TTAAGTRSTT
Mox1	M01443	98	NNNGTAATTANNYNAG
IRXB3	M01377	95	NANNTACATGTANNWNT
HNF1	M00206	95	DGTTAATKAWTNACCAM
Otx2	M01387	94	NNNRGGGATTAATNNNN
Nkx3A	M01383	94	NNNNAAGTACTTAAANN
HOXD3	M01338	93	YTGNNNTAATTANNNN
HMX1	M01481	93	NNNAGCAATTAANGNNN
Brachyury	M00150	93	MKHNTSACACCTAGGTGTGAAATT
TBP	M00471	92	TATAAATW
Pbx-1	M01017	92	NNATCAATCANN
MRF-2	M00454	89	AWSCACAATACNVA
Lhx5	M01353	88	NNNATTAATTAAAWNNN
lmx1b	M01363	87	NNWWWTTAATTAATNNN
Evi-1	M00080	86	AGATAAGATAA
Max	M00119	84	NNANCACGTGNTNN
HOXC9	M01416	83	NNNGGTCATTAANNNN
STAT3	M00225	82	NGNNATTTCCSGGAARTGNNN
HOXA6	M01392	82	AMGKTAATTACCNNAN
AIRE	M01000	82	GGTTATTAATTGGTTATATTGGTTD
HoxB5	M01319	81	ANGNTAATTANCNNAN
HOXC4	M01369	80	CNNNTTAATTAANNANN
Barx1	M01340	80	MNRNYAATTRSYNNAW
HOXA5	M01452	79	ANGNTAATTANCNNAN
Lhx3	M01471	78	NNAATTAATTAAWWNNN
HOXC-8	M01321	78	NNNNNGTAATTANNNT
DMRT2	M01147	77	NAWWTTGWTACATTGW
HoxA2	M01402	76	ANGGTAATTANCNNAN
Brn-3c	M01408	76	NNTTATTAATKANKNC
DMRT3	M01148	75	NWWTTGWTACAWTKT
PPARgamma:RXRalpha,	M00515	73	AAGTAGGTCACNGTGACCYACTT
HOXA1	M01487	73	NTNAGCTAATTAMNNY
Lhx4	M01421	70	NANNCTAATTAGNTWTG
ipf1	M01438	70	NNRNTAATTAGYNCAN
En-1	M01365	69	NNNAACTAATTARKNN

Dlx-1	M01439	69	NNGNNNTAATTANY
Vax-2	M01327	67	NNNNACTAATTANNNC
Nkx3-1	M00451	67	NWATAAGTATWT
Prop-1	M01320	66	NNNNTTAATTAANNNNC
ATF2:c-Jun	M00041	65	TGACGTYA
HSF1	M00146	64	RGAANRTTCN
Brn-4	M01473	64	NNNTAATTAATTANNNN
Pbx-1b	M00124	63	NNCATCAATCAANNW
Cdc5	M00478	63	GATTTAACATAA
HOXA4	M01370	59	NNTNATTAATTAACKNG
Barx-2	M01431	59	TAANTAATTANTKNTN
PMX2B	M01356	58	NNNAATTAATTAANNNG
LH-2	M01325	58	WNNACTAATTAGNNNNN
K-2b	M01348	55	NNAACTAATTARYTNNT
Msx-1	M01412	54	NNNNANTAATTANTNN
ALX-3	M01355	51	WNNAYTAATTARYTNNN
CART1	M01453	49	NNMNTTAATTAANNNNN
Cdx-2	M00729	48	ANANTTTTATKRCC
